# Statistical methods for classification of 5hmC levels based on the Illumina Inifinium HumanMethylation450 (450k) array data, under the paired bisulfite (BS) and oxidative bisulfite (oxBS) treatment

**DOI:** 10.1371/journal.pone.0218103

**Published:** 2019-06-13

**Authors:** Alla Slynko, Axel Benner

**Affiliations:** 1 Department of Statistics and Actuarial Science, University of Waterloo, Waterloo, Canada; 2 Division of Biostatistics, German Cancer Research Center, Heidelberg, Germany; Centre de Recherche en Cancerologie de Lyon, FRANCE

## Abstract

Hydroxymethylcytosine (5hmC) methylation is a well-known epigenetic mark that is involved in gene regulation and may impact genome stability. To investigate a possible role of 5hmC in cancer development and progression, one must be able to detect and quantify its level first. In this paper, we address the issue of 5hmC detection at a single base resolution, starting with consideration of the well-established 5hmC measure Δ*β* and, in particular, with an analysis of its properties, both analytically and empirically. Then we propose several alternative hydroxymethylation measures and compare their properties with those of Δ*β*. In the absence of a gold standard, the (pairwise) resemblance of those 5hmC measures to Δ*β* is characterized by means of a similarity analysis and relative accuracy analysis. All results are illustrated on matched healthy and cancer tissue data sets as derived by means of bisulfite (*BS*) and oxidative bisulfite converting (*oxBS*) procedures.

## Introduction

DNA methylation is known to play a crucial role in the development of diseases such as diabetes, schizophrenia, and some forms of cancer; for details see, e.g., [1–7] and references therein. In order to address the possible impact of DNA methylation on the various biological functions and processes, an entire strand of extensive biological, bioinformatical, and statistical analyses has been developed in the past years. Some of those analyses, most relevant for our setting, were discussed in [8–15]. A substantial part of the methods introduced in those analyses aims at quantifying the actual level of DNA methylation, in particular on a single nucleotide resolution in genomic DNA.

At some point, this research indicated that the obtained DNA methylation level, sometimes referred to as “total DNA methylation” [16, 17], can be split, inter alia, into 5-hydroxymethylcytosine (5hmC) and 5-methylcytosine (5mC) components, with 5mC playing an important role in gene silencing and genome stability [18]. The second component, 5hmC methylation, was first discovered in 2009 as another form of cytosine modification [19–21]. Since then, its function as an intermediate in active DNA demethylation and an important epigenetic regulator of mammalian development which is strongly associated with genes and regulatory elements in the genome, as well as its role as a possible epigenetic mark impacting genome stability has come into the spotlight [16, 18, 22–38]. At that point, the questions concerning reliable identification and accurate quantification of 5hmC levels emerged.

Until now, a number of techniques for the quantification of 5hmC levels have been established [16–18, 24, 28, 31, 39–41]. Two key techniques to be named here are the TET-assisted bisulfite (*TAB*) technique and the oxidative bisulfite (*oxBS*) technique. The *TAB* technique is based on the conversion of 5mC to 5hmC in mammalian DNA by means of TET emzymes [17, 31]. When using the *oxBS* technique, 5hmC methylation levels can be obtained by means of the paired bisulfite sequencing (*BS*) and oxidative bisulfite sequencing (*oxBS*) procedures [18]. In particular, since the *BS* procedure can only differentiate between methylated and unmethylated cytosine bases, and cannot discriminate between 5mC and 5hmC, the *oxBS* procedure must be applied, in order to determine the level of 5hmC at a considered nucleotide position. This procedure yields Cs only at 5mC sites while oxidating 5hmC to 5-formylcytosine (5fC) and later converting them to uracil. As a result, an amount of 5hmC at each particular nucleotide position can be determined as the difference between the *oxBS* (which identifies 5mC) and the *BS* (which identifies 5mC+5hmC) readouts. In the present paper, all obtained results are illustrated on paired *BS* and *oxBS* data.

In order to quantify the 5hmC level in the context of the *oxBS* technique, and, in particular, to identify a given CpG site as being either hydroxymethylated or non-hydroxymethylated, the following quantity was introduced in [41]

$$\begin{array}{c} \Delta \beta^{oxBS}=\beta_{BS}-\beta_{oxBS}=\frac{M_{BS}}{M_{BS}+U_{BS}+100}-\frac{M_{oxBS}}{M_{oxBS}+U_{oxBS}+100}. \end{array}$$ (1)

Here, *M* denotes the intensity of the methylated allele, *U* is the intensity of the unmethylated allele, *β*_*BS*_ is the methylation level obtained from the *BS* method, and *β*_*oxBS*_ is the methylation level derived by means of the *oxBS* method. As stated in [31, 41], the quantity Δ*β*^*oxBS*^ computed for each single CpG and sample can be interpreted as a “measure of hydroxymethylation” and “a reflection of the 5hmC level at each particular probe location”. This measure can then be applied in the screening step so as to exclude from further analysis those CpGs that do not appear to be hydroxymethylated.

In [42], the authors introduced a related quantity Δ*m*^*oxBS*^, defined as a difference of the corresponding *m*-values [13], to be another measure for identification and quantification of the 5hmC levels. However, our discussion in S1 Appendix shows that in the context of the 5hmC identification both measures, Δ*β*^*oxBS*^ and Δ*m*^*oxBS*^, flag exactly the same cytosines as being substantially hydroxymethylated and thus can be used *interchangeably*.

Due to its definition, Δ*β*^*oxBS*^ in (1) can take values between -1 and 1, with negative values of Δ*β*^*oxBS*^ representing “false differences in methylation score between paired *BS*-only and *oxBS* data sets” and being interpreted as a “background noise” [41].

While applying Δ*β*^*oxBS*^ for the identification of substantially hydroxymethylated cytosines, the issue of an appropriate Δ*β*^*oxBS*^ *threshold* arises; such threshold can be applied “to identify a probe-set of substantially hydroxymethylated cytosines”. In [41], the threshold for Δ*β*^*oxBS*^ has been set to 0.3 or 30%. However, it is not evident, whether such threshold can be applied for any given data set or should be specified for each particular setting.

This paper is organized as follows. First, we address the applicability of the 5hmC measure Δ*β*^*oxBS*^ (in the following notation just Δ*β*) for detection of hydroxymethylated CpGs and then indicate several limitations of this measure by discussing its properties, both analytically and on data sets. Further, we propose several alternative hydroxymethylation measures which can also be applied for the 5hmC identification and compare their properties and resemblance with those of Δ*β*. Relative accuracy and resemblance of all three considered 5hmC measures are discussed numerically, under the assumption that no gold standard is available. All data analyses were performed on 38 matched samples, with cancer and healthy tissue available for each sample.

## Discussion

### On the applicability of Δ*β* for 5hmC detection

According to [8], for a given methylated and unmethylated intensities *M* and *U*, the methylation level of the particular probe can be described by the *methylation proportion*

$$\begin{array}{c} \beta =\frac{M}{M+U+100}. \end{array}$$ (2)

Thus, the 5hmC measure Δ*β*^*oxBS*^ in (1) is just the difference of two methylation proportions as derived from *BS* and *oxBS* treatment, respectively. This simple definition, while appearing to be plausible at first, nevertheless leads to a number of ambiguities as discussed below.

The first ambiguity arising from (1) concerns the application of Δ*β* as a measure for the identification of hydroxymethylated CpGs, and, in particular, its adequate interpretation as such. Even if both components in the difference (1) do represent the respective methylation proportions for *BS* and *oxBS* data, these proportions are evidently calculated on two different bases: the proportion *β*_*BS*_ represents the methylation proportion based on the global *BS* intensity *M*_*BS*_ + *U*_*BS*_, whereas the proportion *β*_*oxBS*_ represents the methylation proportion based on the global *oxBS* intensity *M*_*oxBS*_ + *U*_*oxBS*_. Thus, a direct comparison of these two proportions is difficult to justify and, as a result, the interpretation of Δ*β* as “a reflection of the 5hmC level at each particular probe” suggested in [41] is not well founded.

**Fig 1:**
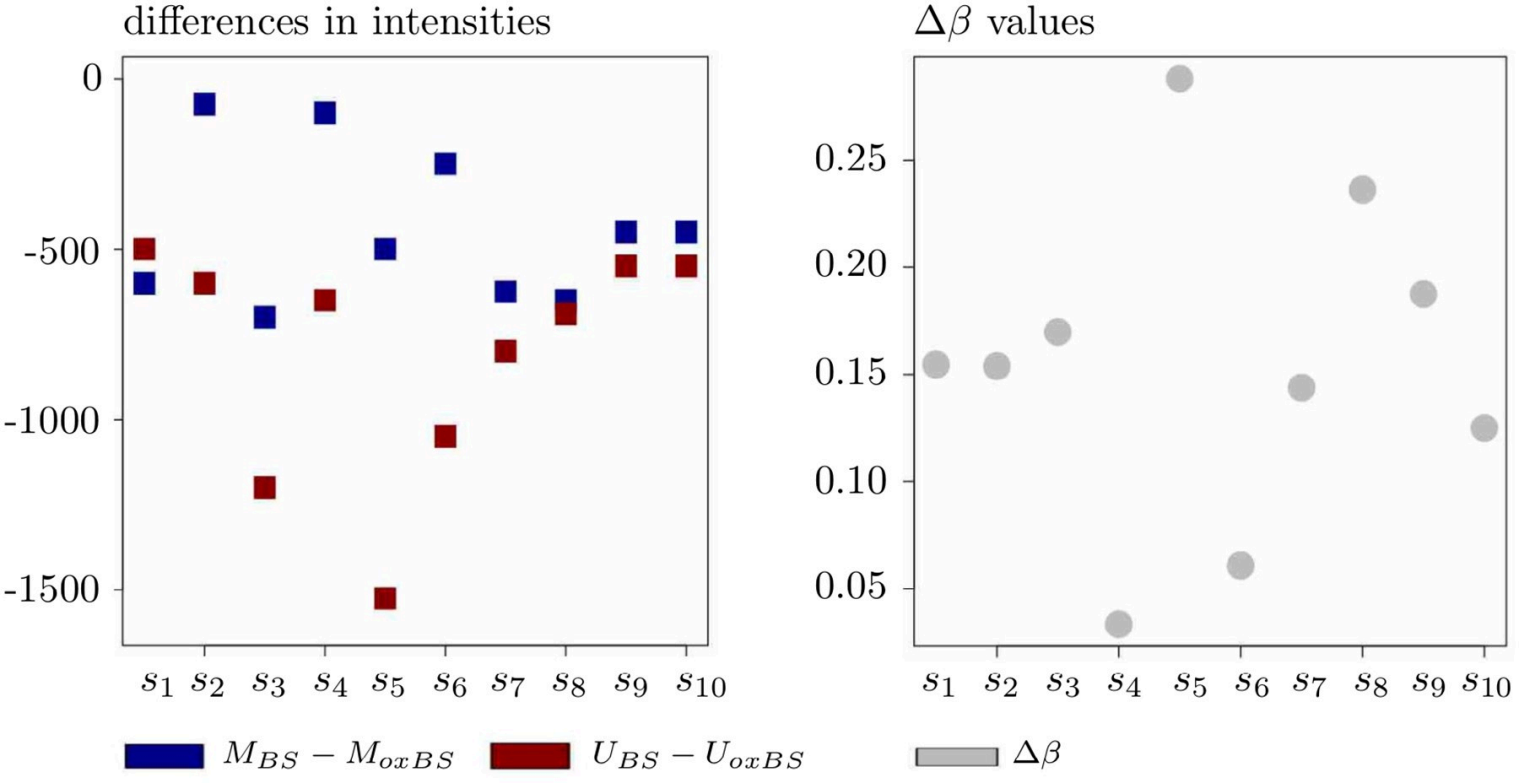
On the interpretation of Δ*β* as a 5hmC measure in case with Δ*β* > 0. Negativity of the differences on the left-hand panel implies that none of the data points *s*_1_, *s*_2_, …*s*_10_ shows any substantial 5hmC level, but, due to Δ*β* > 0, all these points will nevertheless be flagged by Δ*β* as being hydroxymethylated.

Further, while identifying hydroxymethylated CpGs in the context of the screening step, the outcomes of Δ*β* are interpreted as follows [41]: Positive values of Δ*β* are taken as an indicator for a substantial 5hmC level and “represent potential sites of 5hmC”, whereas small values of Δ*β* should indicate no or only nonsubstantial hydroxymethylation levels. Negative values of Δ*β* are considered as resulting from background noise; for the 5hmC measure Δ*m*, the same view is shared in [42]. To analyze this interpretation, let us first refer to Fig 1. As the left-hand panel of that figure shows, all ten simulated data points *s*_1_, *s*_2_, …, *s*_10_ satisfy both conditions

$$\begin{array}{c} M_{BS}^{i}<M_{oxBS}^{i}\;\;\;\text{and}\;\;\;U_{BS}^{i}<U_{oxBS}^{i},\;\;\;\;\;\;\;\;\;i=1,2,\ldots ,10 \end{array}$$ (3)

simultaneously which intuitively should be interpreted as “no substantial 5hmC level observed”. Nevertheless, the condition Δ*β* > 0 holds for each of these ten data points as well; see the right-hand panel of Fig 1 for an illustration.

**Fig 2:**
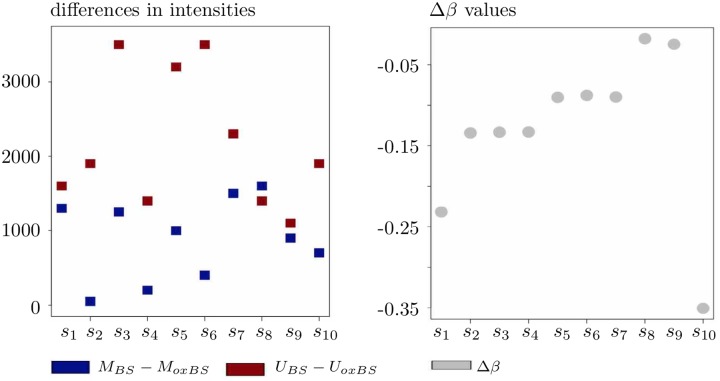
On the interpretation of Δ*β* as a 5hmC measure in case with Δ*β* < 0. Due to the positivity of the differences on the left-hand panel, all ten data points *s*_1_, *s*_2_, …, *s*_10_ appear to exhibit a substantial level of 5hmC, whereas the right-hand panel shows negative Δ*β* values.

Further, the left-hand panel of Fig 2 introduces another ten simulated data points *s*_1_, *s*_2_, …*s*_10_ that satisfy both

$$\begin{array}{c} M_{BS}^{i}>M_{oxBS}^{i}\;\;\;\text{and}\;\;\;U_{BS}^{i}>U_{oxBS}^{i},\;\;\;\;\;\;\;\;i=1,2,\ldots ,10. \end{array}$$ (4)

At the same time, the condition Δ*β* < 0 holds for each of *s*_1_, *s*_2_, …*s*_10_ as well; see the right-hand panel of Fig 2 for an illustration. Thus, even though the data points *s*_1_, *s*_2_, …*s*_10_ in Fig 2 actually appear to exhibit a substantial 5hmC level due to their *BS* intensities exceeding their *oxBS* intensities, they will not be selected by the measure Δ*β* as being hydroxymethylated.

**Fig 3:**
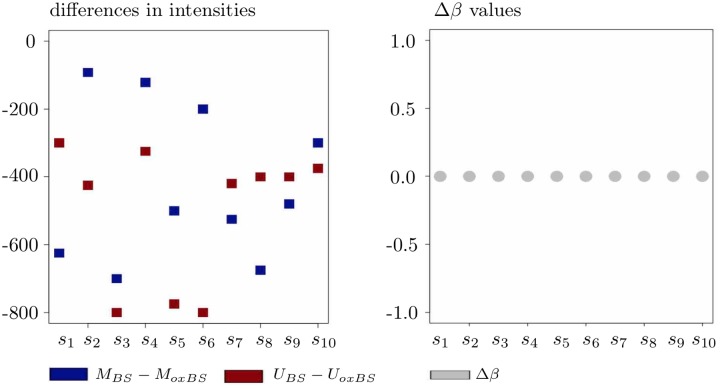
On the interpretation of Δ*β* as a 5hmC measure in case with Δ*β* = 0. Negativity of the differences on the left-hand panel implies that none of the data points should show any substantial 5hmC level.

One of the main advantages of the measure *β*, which has definitely contributed to its common application as a methylation measure, is its intuitive interpretation as an approximation of the *percentage of methylation* [13]; thereby *β* = 0 indicates unmethylated probes and *β* = 1 denotes fully methylated probes. Unfortunately, this interpretation does not carry over to the measure Δ*β*. Indeed, in (1) the condition Δ*β* = 0 solely implies

$$\begin{array}{c} \frac{M_{BS}}{M_{oxBS}}=\frac{U_{BS}+100}{U_{oxBS}+100} \end{array}$$ (5)

and it is unclear how this last equality should be interpreted in terms of the observed 5hmC level. In particular, Fig 3 demonstrates that we can obtain Δ*β* = 0 in cases with “no substantial 5hmC level observed”, i.e., in cases where the conditions

$$\begin{array}{c} M_{BS}^{i}<M_{oxBS}^{i}\;\;\;\text{and}\;\;\;U_{BS}^{i}<U_{oxBS}^{i},\;\;\;\;\;\;\;\;\;i=1,2,\ldots ,10 \end{array}$$ (6)

hold. Similar results can be derived in cases with “a substantial 5hmC level observed”, i.e., in cases with

$$\begin{array}{c} M_{BS}^{i}>M_{oxBS}^{i}\;\;\;\text{and}\;\;\;U_{BS}^{i}>U_{oxBS}^{i},\;\;\;\;\;\;\;\;\;i=1,2,\ldots ,10. \end{array}$$ (7)

Altogether, our analyses of the conditions Δ*β* > 0, Δ*β* = 0, and Δ*β* < 0 show that their interpretations as indicators for substantial hydroxymethylation, no hydroxymethylation, and background noise may become problematic in certain situations.

Another ambiguity arising from (1) is related to the choice of the number 100 in the denominators *M*_*BS*_ + *U*_*BS*_ + 100 and *M*_*oxBS*_ + *U*_*oxBS*_ + 100 of the expression (1) for Δ*β*. This choice seems to stem from the practical convention in the definition of *β* values [13], and just being transferred at the definition of Δ*β* [31, 41]. As a matter of fact, there is no strong reason why the correction term 100 in the denominator of (2) should not be replaced with any other value *α* > 0. In fact, such replacement would lead to the following more general definition of the methylation proportion

$$\begin{array}{c} \beta \left(\alpha \right)=\frac{M}{M+U+\alpha },\;\;\;\text{with}\;\;\;\alpha >0. \end{array}$$ (8)

**Fig 4:**
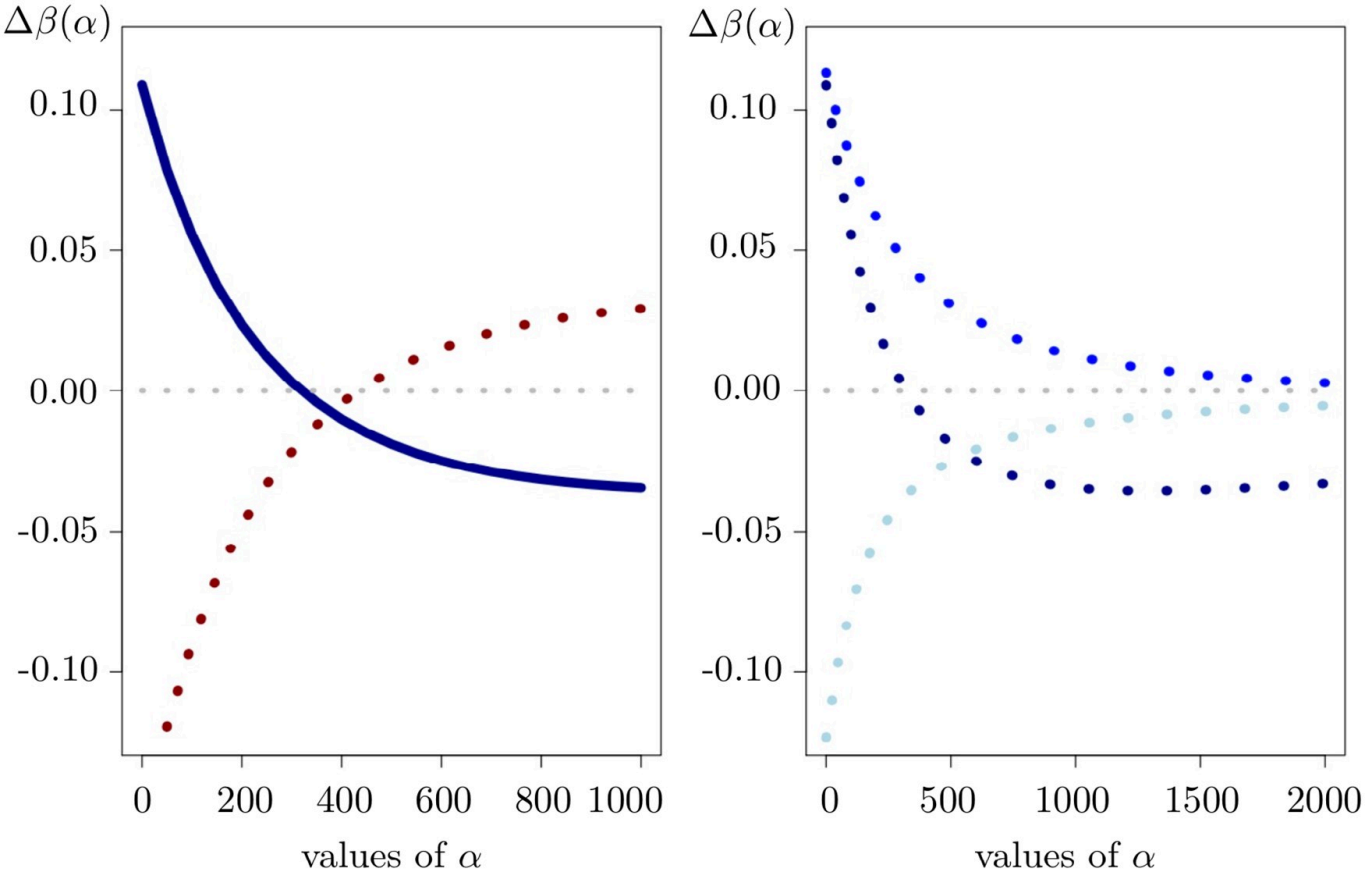
Sign change and convergence of the 5hmC measure Δ*β*(*α*). The left-hand panel: Δ*β*(*α*) changing its sign from positive to negative (the dark blue curve, healthy tissue) and from negative to positive (the dark red dotted curve, cancer tissue) as *α* increases. The result refers to a given CpG (*cg*00050873) and sample (*sample 7*). The right-hand panel: Convergence of Δ*β*(*α*) for healthy tissue, a given sample (*sample 7*) and three CpGs (*cg*00050873, *cg*05480730, *cg*10698069).

While one can safely argue that the actual choice of the parameter *α* is not crucial for the interpretation of the methylation proportion *β*(*α*) itself [13], this choice may become critical when using the sign of the measure

$$\begin{array}{c} \Delta \beta \left(\alpha \right)=\beta_{BS}\left(\alpha \right)-\beta_{oxBS}\left(\alpha \right)=\frac{M_{BS}}{M_{BS}+U_{BS}+\alpha }-\frac{M_{oxBS}}{M_{oxBS}+U_{oxBS}+\alpha }, \end{array}$$ (9)

as an indicator for hydroxymethylation in the screening step. In particular, under certain conditions, the sign of Δ*β*(*α*) can change from positive to negative or vice versa as *α* varies; see the left-hand panel of Fig 4 as well as Fig A in S1 Appendix for an illustration.

**Fig 5:**
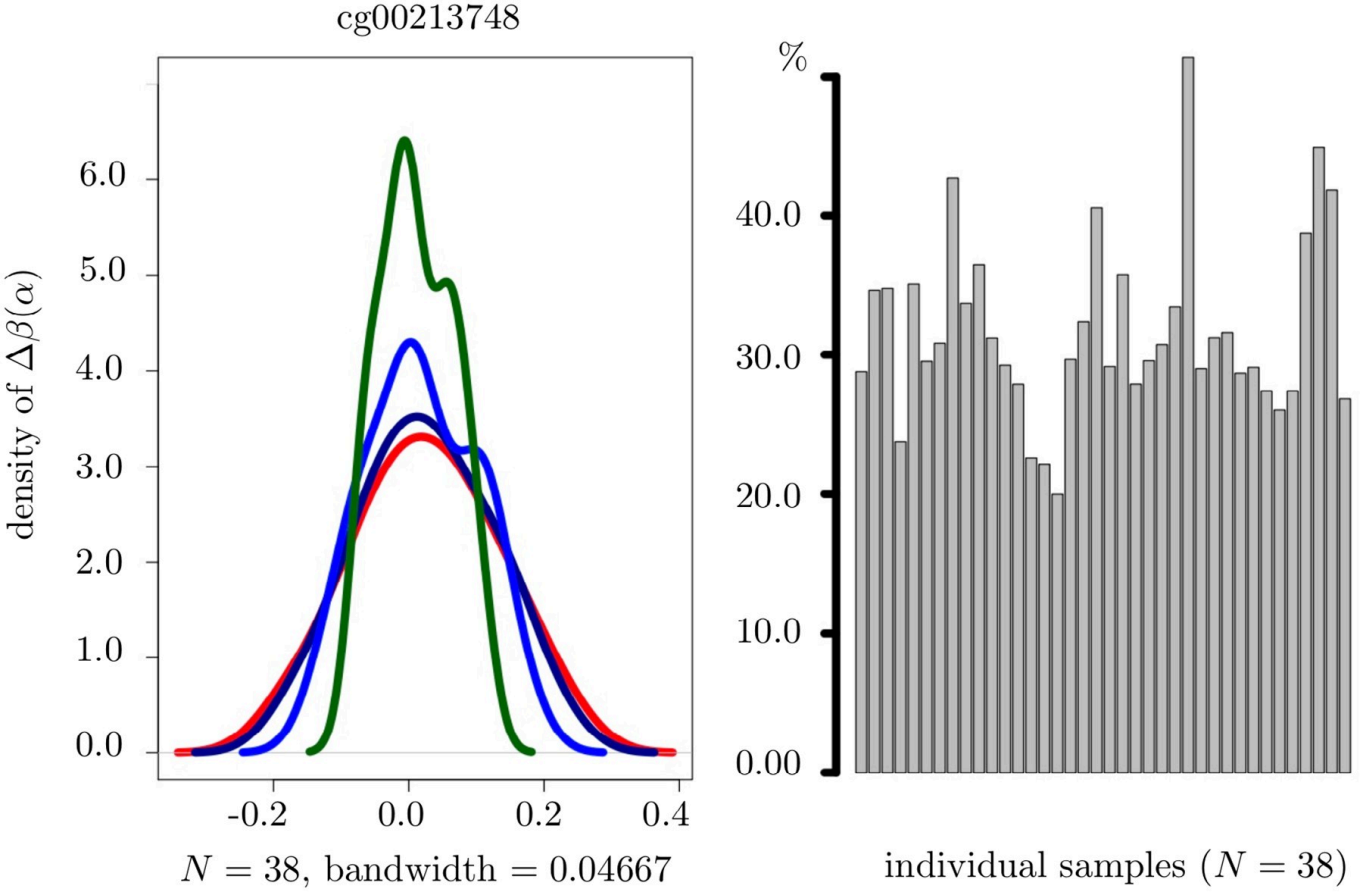
Density of Δ*β*(*α*) and the percentage of CpGs, where Δ*β*(*α*) may change its sign for varying values of *α*. The left-hand panel: Density of Δ*β*(*α*), for a given CpG (*cg*00213748) and *α* = 0, 100, 500 and 2000 (the red, the dark blue, the blue and the dark green curves, respectively). The right-hand panel: The percentage of CpGs, where Δ*β*(*α*) may change its sign for varying values of *α*. All results were computed on cancer tissue and across all 38 samples.

Further, Fig 5 shows changes in the density of Δ*β*(*α*) as well as the percentage of CpGs (for each given sample) where Δ*β*(*α*) may change its sign as *α* increases.

In view of such dependence of Δ*β*(*α*) on the choice of *α*, a question concerning the possible impact of this choice on the percentage of CpGs satisfying the condition Δ*β*(*α*) > 0 and thus identified as being hydroxymethylated at the end of the screening step arises.

### Alternative 5hmC measures

One of the limitations of the 5hmC measure Δ*β*(*α*) we discussed in the previous section concerns its interpretation and robustness with respect to the choice of the correction term *α*. To overcome this limitation, we now introduce two alternative measures which can be used in the screening procedure while indicating CpGs with a substantial level of 5hmC; the basic properties of these measures are discussed in S2 Appendix.

We start our analysis by considering the behavior of Δ*β*(*α*) (and also Δ*m*(*α*) as discussed in S2 Appendix) for increasing values of *α*. As follows from (9), Δ*β*(*α*) vanishes as *α* increases; see the right-hand panel of Fig 4 for an illustration. This convergence result is also transferable to Δ*m*(*α*), with the only difference that in case of Δ*β*(*α*) the limit will always be zero, independently of the CpGs, sample, and the tissue chosen, whereas in case of Δ*m*(*α*) the limit depends on the CpG, sample, and tissue under consideration.

The convergence results for Δ*β*(*α*) and Δ*m*(*α*) imply that the percentage of CpGs satisfying the condition Δ*β*(*α*) > 0 and Δ*m*(*α*) > 0 for a given sample, respectively, approaches a positive constant as *α* increases. Standard computations verify this limit value to be just the percentage of CpGs satisfying *M*_*BS*_ > *M*_*oxBS*_ for a given sample; see S2 Appendix for details.

Inspired by the convergence results obtained for the measures Δ*β*(*α*) and Δ*m*(*α*), we next propose

$$\begin{array}{c} \Delta m^{\infty }=\log_{2}\frac{M_{BS}}{M_{oxBS}}\left(=\lim_{\alpha ↑\infty }\Delta m\left(\alpha \right)\right) \end{array}$$ (10)

as the first alternative 5hmC measure that can be used for the detection of hydroxymethylated CpGs. Note that Δ*m*^∞^ is well-defined for all CpGs satisfying *M*_*BS*_ > 0 and *M*_*oxBS*_ > 0 simultaneously.

The main advantage of the measure Δ*m*^∞^ in comparison to the measures Δ*β*(*α*) and Δ*m*(*α*) is its complete independence of the correction term *α*; this fact makes Δ*m*^∞^ more robust for application in the screening step. Furthermore, the sign of Δ*m*^∞^ has a very intuitive interpretation. Indeed, we get Δ*m*^∞^ > 0 if *M*_*BS*_ > *M*_*oxBS*_ holds, i.e., if the global methylated intensity *M*_*BS*_ exceeds the “adjusted” methylated intensity *M*_*oxBS*_. In all other cases we will have Δ*m*^∞^ ≤ 0; for instance, Δ*m*^∞^ = 0 implies *M*_*BS*_ = *M*_*oxBS*_, which can intuitively be interpreted as “no substantial 5hmC level observed”.

In the context of our screening procedure, the most crucial question concerns a relation between the subsets of CpGs satisfying Δ*m*(*α*) > 0 and Δ*m*^∞^ > 0, respectively. To answer this question in a formal way, we divided the set of all CpGs with Δ*m*(*α*) > 0 in several disjoint subsets, and showed that, for a given sample and increasing *α*, the union of these subsets converges to the subset of CpGs satisfying *M*_*BS*_ > *M*_*oxBS*_; see S2 Appendix for more details.

Due to its definition, Δ*m*^∞^ does not take into account the unmethylated intensities *U*_*BS*_ and *U*_*oxBS*_. This may become an issue even if the role of these intensities in the detection of hydroxymethylated CpGs has not been clarified yet. We address this issue by proposing another measure for selecting CpGs with a substantial level of hydroxymethylation, namely,

$$\begin{array}{c} \Delta h=1-\frac{M_{oxBS}+U_{oxBS}}{M_{BS}+U_{BS}}. \end{array}$$ (11)

In (11), *M*_*BS*_ + *U*_*BS*_ is the global intensity obtained from the *BS* procedure and *M*_*oxBS*_ + *U*_*oxBS*_ is the global intensity derived by means of the *oxBS* procedure.

For CpGs with *M*_*BS*_ + *U*_*BS*_ exceeding *M*_*oxBS*_ + *U*_*oxBS*_, i.e., for those CpGs which can be intuitively interpreted as exhibiting a substantial level of hydroxymethylation, the measure Δ*h* must range between 0 and 1. In particular, the values of Δ*h* close to zero correspond to *M*_*BS*_ + *U*_*BS*_ being approximately equal to *M*_*oxBS*_ + *U*_*oxBS*_ and thus the global 5hmC level being (almost) negligible. On the other hand, for Δ*h* approximately equal to one we deduce that *M*_*oxBS*_ + *U*_*oxBS*_ must be substantially smaller than *M*_*BS*_ + *U*_*BS*_ and thus the global 5hmC level has to be high. Altogether, larger values of Δ*h* correspond to larger proportions of the global 5hmC levels and we can interpret Δ*h* as *the proportion of 5hmC in the global methylation*.

Our intuition in the interpretation of the values of Δ*h* is based on the assumption that a substantial 5hmC level is associated with a substantial decrease in the overall intensities *M* + *U*, with *M*_*BS*_ + *U*_*BS*_ > *M*_*oxBS*_ + *U*_*oxBS*_ for a given CpG site. Such interpretation is induced by the fact that, in contrast to the methylation process, a role of the unmethylated intensities *U* in the hydroxymethylation process is unclear. Thus, negative values of Δ*h* are currently treated as a measurement error. Note that in (11) one has to assume that *M*_*BS*_ + *U*_*BS*_ is different from zero; in other words, all CpGs with *M*_*BS*_ + *U*_*BS*_ equal to zero have to be excluded from the analysis as exhibiting measurement error.

In view of the screening procedure, we also analyzed whether positive values of the measure Δ*h* lead to positivity of other 5hmC measures introduced above, and vice versa. As (11) implies, the inequality Δ*h* > 0 holds for

$$\begin{array}{c} M_{BS}+U_{BS}>M_{oxBS}+U_{oxBS}. \end{array}$$ (12)

However, the latter inequality is not sufficient to make a statement about the sign of the measures Δ*β*(*α*) and Δ*m*^∞^, so that additional assumptions are needed; see S2 Appendix for details.

Altogether, our discussion indicates that the application of Δ*h* for the detection of hydroxymethylated CpGs can be of advantage, since this 5hmC measure overcomes the limitation of both 5hmC measures considered earlier. In particular, this measure does not depend on the choice of the correction term *α*, has an intuitive interpretation of its outcomes in terms of the observed 5hmC level, and can be computed directly from measured array data.

## Materials and methods

### Numerical analyses of the resemblance of Δ*β*(*α*), Δ*m*^∞^ and Δ*h*

In the previous sections we considered three 5hmC measures, Δ*β*(*α*), Δ*m*^∞^ and Δ*h*, as possible tools for the classification of CpGs into hydroxymethylated and those which do not exhibit a substantial level of hydroxymethylation. To estimate a possible classification error, one would usually compare each of these 5hmC measures with a certain gold standard. However, no gold standard is available in our case, since even the actual meaning of the formulations “a substantial 5hmC level observed” or “no substantial 5hmC level observed” in terms of measured methylated and unmethylated intensities *M* and *U* is unclear so far. One of possible ways to evaluate the accuracy of Δ*β*(*α*), Δ*m*^∞^ and Δ*h* in the *absence of a gold standard*, as proposed in this section, is to describe this accuracy in terms of relative sensitivities and specificities of these measures with respect to each other. On the other hand, the resemblance of the considered 5hmC measures with respect to each other can also be addressed by means of a similarity analysis.

Numerical analyses of the present section were motivated by the discussions presented in [24, 43–48].

### Study cohort, 5hmC isolation, data preprocessing

All analyses were performed on 38 paired samples, with both (colorectal) cancer and normal tissue available for each sample. All 38 patients were enrolled in the ongoing population-based case-control study DACHS (Darmkrebs: Chancen der Verhütung durch Screening, http://dachs.dkfz.org/dachs/), extensively described in [49]. Data collecting and patient recruitment procedures as well as the processes of DNA isolation and methylation profiling using the Infinium HumanMethylation450 BeadChip array (Illumina) are similar to those described in [50]. All data are publicly available at https://zenodo.org/record/2639285#.XLYzNKZS_XE.

All data analyses were performed using the computational environment R, V.3.5.2 (http://www.r-project.org/). Raw data signals from each of the *BS-* and *oxBS-* converted samples were preprocessed using the R/Bioconductor *minfi*-package [51]. In particular, the procedure *preprocessRaw* from that package was applied in order to convert the red/green channel for an Illumina methylation array into methylation signal.

## Results

### Prevalence of positive results

**Fig 6:**
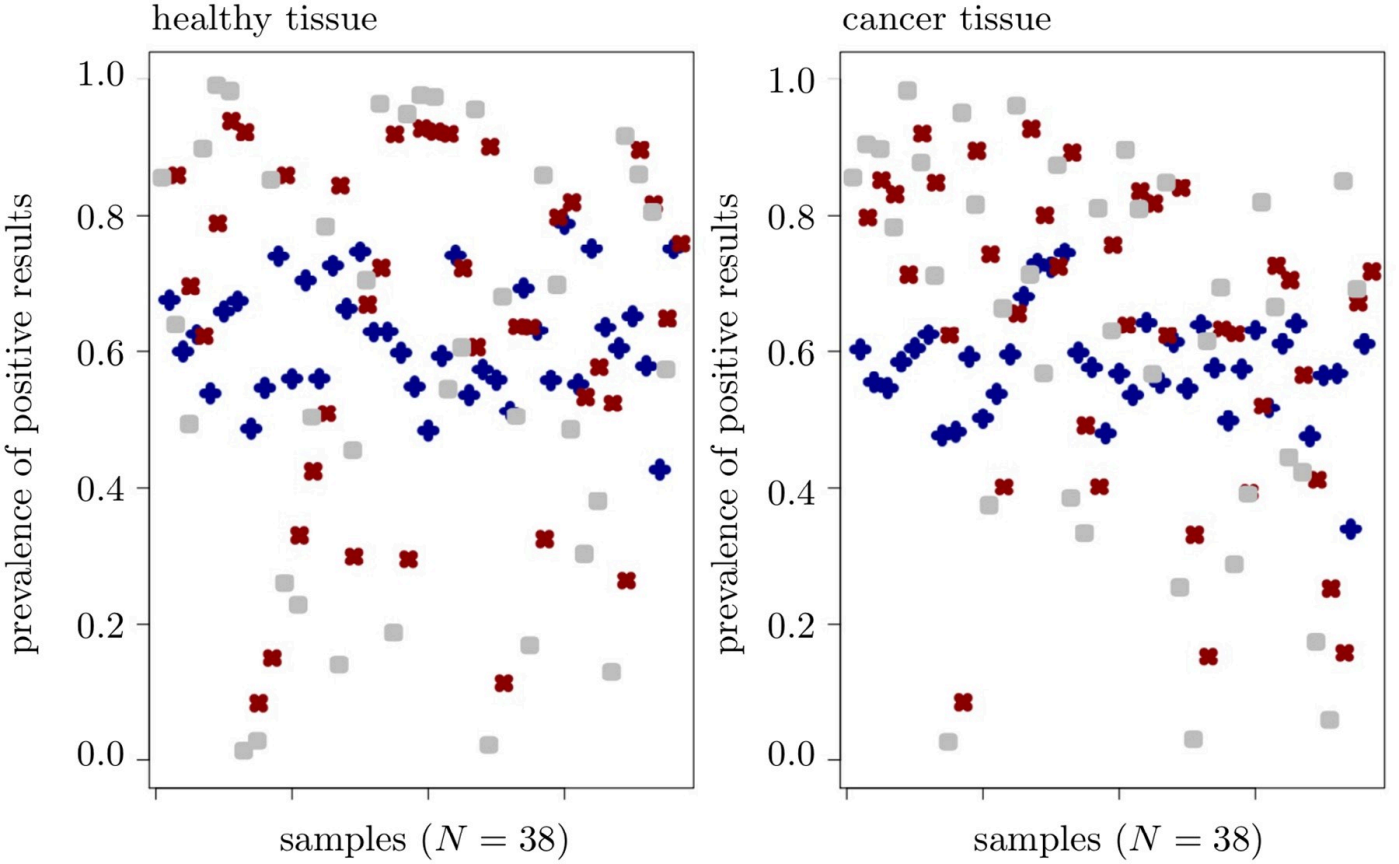
Sample-wise prevalence of positive results. The dark blue dots correspond to the percentage of CpGs with Δ*β*(100) > 0, the dark red dots to Δ*m*^∞^ > 0 and the grey dots to Δ*h* > 0.

We applied all three considered 5hmC measures to both healthy and cancer tissue and computed the percentage of CpGs satisfying Δ*β*(100) > 0, Δ*m*^∞^ > 0 and Δ*h* > 0 for each given sample; Fig 6 illustrates the obtained results. Note that such *prevalence of positive results* is crucial in the screening procedure and represents the most intuitive approach for the comparison of any two 5hmC measures. Dependence of the prevalence of positive results of the measure Δ*β*(*α*) on the choice of *α* is discussed in S1 Appendix.

Further, we adopted the statement in [24] on a reduction of 5hmC levels in cancer tissue to the prevalence of positive results, by expecting this prevalence to be higher in healthy tissue compared to cancer one. In a sample-wise analysis, this anticipation was indeed confirmed for the 5hmC measure Δ*β*(100), but not for the measures Δ*m*^∞^ and Δ*h*.

The same analysis, performed CpG-wise, i.e., with prevalence of positive results computed for each single CpG across all 38 samples that provides the *hydroxymethylation level* for each given CpG, again showed a significant reduction in 5hmC levels as obtained on cancer tissue, in particular for the measures Δ*β*(100) and Δ*m*^∞^. Contrary to our expectations, for the measure Δ*h*, prevalence of positive results was significantly lower in healthy tissue compared to cancer one.

Next, we compared prevalences of positive results of any two 5hmC measure on a given tissue, in order to investigate the *conservativeness* of these measures when screening for hydroxymethylated CpGs. This analysis, performed sample-wise, resulted in the 5hmC measure Δ*m*^∞^ being less conservative than Δ*h* on healthy tissue and less conservative than Δ*β*(100) on cancer tissue; see Fig 6 for an illustration. The same analysis, performed CpG-wise, determined Δ*m*^∞^ as being the least conservative 5hmC measure, on both considered tissues. Further, on healthy tissue Δ*β*(100) appeared to be less conservative than Δ*h*, whereas on cancer tissue, Δ*h* was less conservative than Δ*β*(100). This result can be interpreted as an evidence of the *tissue effect* [41, 52].

We also analyzed the *joint prevalence of positive results* defined as the percentage of CpGs with any two 5hmC measures being positive; such joint prevalence characterizes the *agreement* between any two 5hmC measures in the context of the screening step. Sample-wise analysis did not reveal any significant differences in these joint prevalences as calculated on healthy and cancer tissue. On the other hand, the joint prevalence of the measures Δ*β*(100) and Δ*m*^∞^ appeared to exceed the joint prevalence of Δ*β*(100) and Δ*h* significantly, on both considered tissues. The same result, again for both tissues, holds for the joint prevalences of the measures Δ*m*^∞^ and Δ*h* as well as of the measures Δ*β*(100) and Δ*h*. Finally, on cancer tissue, the joint prevalence of the measures Δ*β*(100) and Δ*m*^∞^ significantly exceeded the joint prevalence of the measures Δ*m*^∞^ and Δ*h*. In total, we conclude that, in a sample-wise analysis performed on cancer tissue, the 5hmC measures Δ*β*(100) and Δ*m*^∞^ demonstrate the strongest agreement, followed by agreement between the measures Δ*m*^∞^ and Δ*h*.

**Fig 7:**
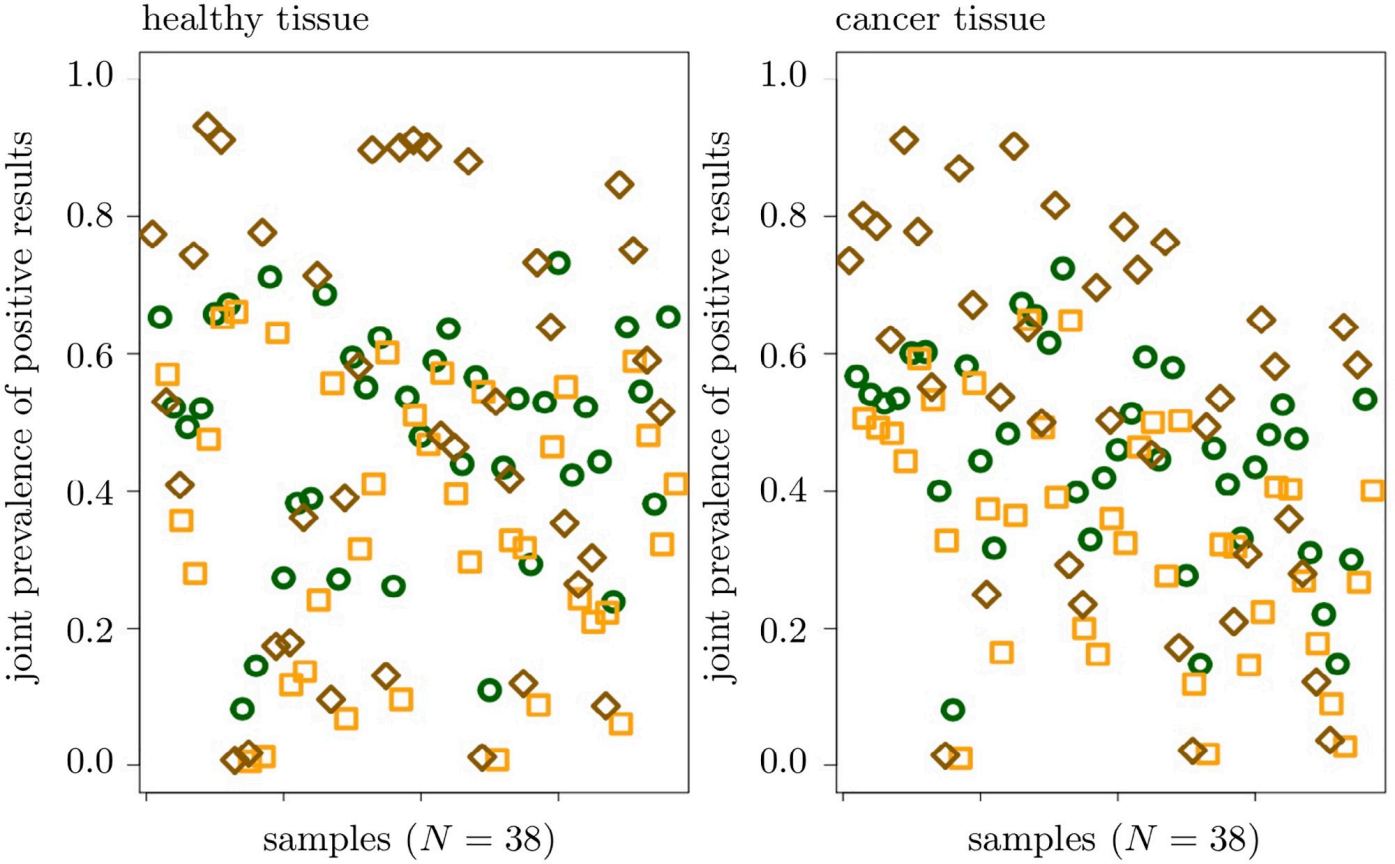
Sample-wise joint prevalence of positive results. Orange squares correspond to the values for Δ*β*(100) and Δ*h*, dark green circles to the values for Δ*β*(100) and Δ*m*^∞^ and brown squares to the values for Δ*h* and Δ*m*^∞^.

**Fig 8:**
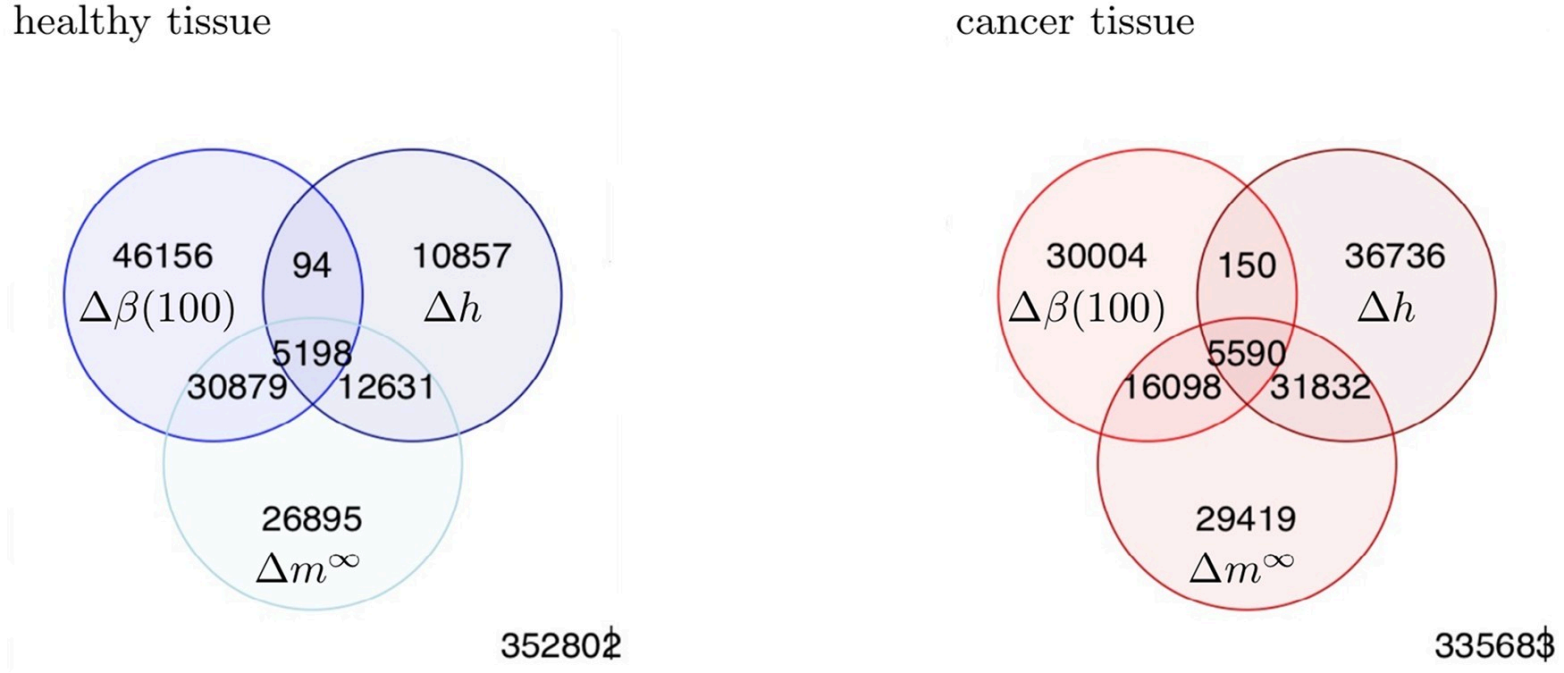
The number of substantially hydroxymethylated CpGs as identified by all three 5hmC measures. The number of substantially hydroxymethylated CpGs as identified by all three 5hmC measures, on healthy (the left-hand panel) and cancer (the right-hand panels) tissues and across all 38 samples. A CpG site is considered to be substantially hydroxymethylated under a given 5hmC measure *x*, if at least 75% of all values of *x* computed for this CpG and across all 38 samples are positive.

The same joint prevalence analysis, performed CpG-wise, revealed the joint prevalence of the measures Δ*β*(100) and Δ*m*^∞^ on healthy tissue being significantly higher than the corresponding joint prevalence on cancer tissue; similar result is true for the joint prevalence of the measures Δ*β*(100) and Δ*h*. As in case of a sample-wise analysis, the joint prevalence of the measures Δ*β*(100) and Δ*m*^∞^ significantly exceeded the joint prevalence of Δ*β*(100) and Δ*h*, both on healthy and cancer tissue; the same relation is true for the joint prevalences of the measures Δ*m*^∞^ and Δ*h* and of the measures Δ*β*(100) and Δ*h*. On the other hand, in contrast to the results of the sample-wise analysis above, the joint prevalence of the measures Δ*β*(100) and Δ*m*^∞^ is significantly lower than the joint prevalence of the measures Δ*m*^∞^ and Δ*h*, both on healthy and cancer tissue. Altogether, the CpG-wise analysis showed the highest agreement between the measures Δ*m*^∞^ and Δ*h*, followed by the agreement between the measures Δ*β*(100) and Δ*m*^∞^; the 5hmC measures Δ*β*(100) and Δ*h* demonstrated the lowest pairwise agreement, consistent with the results of the sample-wise analysis. Sample-wise joint prevalence of positive results is visualized in Fig 7. Joint agreement between all three 5hmC measures is illustrated in Fig 8; for more results see also Figs A and B in S3 Appendix.

To summarize the results of our discussion above, we state that the 5hmC measure Δ*β*(100) demonstrates a higher agreement with Δ*m*^∞^ than with Δ*h*. Moreover, the agreement between the measures Δ*m*^∞^ and Δ*h* exceeds the agreement between Δ*β*(100) and Δ*h*.

### Similarity analyses

In order to address the resemblance of the proposed 5hmC measures without making any statement about their performance, *similarity analyses* can also be applied; the main tool of such analyses is a *similarity coefficient*. There is a variety of similarity coefficients proposed in literature. For an overview see, e.g., [53–56] and references therein.

In order to quantify the *pairwise similarity* of the proposed 5hmC measures Δ*β*(*α*), Δ*m*^∞^, and Δ*h* in the context of the screening step, we first considered the similarity coefficient S, also known as *the simple matching coefficient* [53, 54]. In particular, for a given CpG and two given 5hmC measures *x*_1_ and *x*_2_ we rewrite this similarity coefficient as

$$\begin{array}{c} S\left(x_{1},x_{2}\right)=\frac{1}{n}(\sum_{i=1}^{n}I_{\left\{x_{1}^{i}>0\right\}}I_{\left\{x_{2}^{i}>0\right\}}+\sum_{i=1}^{n}I_{\left\{x_{1}^{i}\leq 0\right\}}I_{\left\{x_{2}^{i}\leq 0\right\}}). \end{array}$$ (13)

Here *n* is the number of samples under consideration, *I*_{*x*>0}_ is the indicator function, with *I*_{*x*>0}_ = 1 for *x* > 0 and *I*_{*x*>0}_ = 0 otherwise, and $x_{j}^{i}$ is the value of the measure *x*_*j*_(*j* = 1, 2) in the *i*th CpG. Clearly, the similarity coefficient S in (13) ranges between 0 and 1, with 1 corresponding to *complete similarity* and 0 to *complete dissimilarity* between the considered two measures *x*_1_ and *x*_2_. Moreover, the similarity coefficient S represents an extension of the prevalence of positive results introduced earlier, since it considers not only the CpG sites that were flagged as hydroxymethylated but also those CpG sites that were identified as non-hydroxymethylated by two considered 5hmC measures.

**Fig 9:**
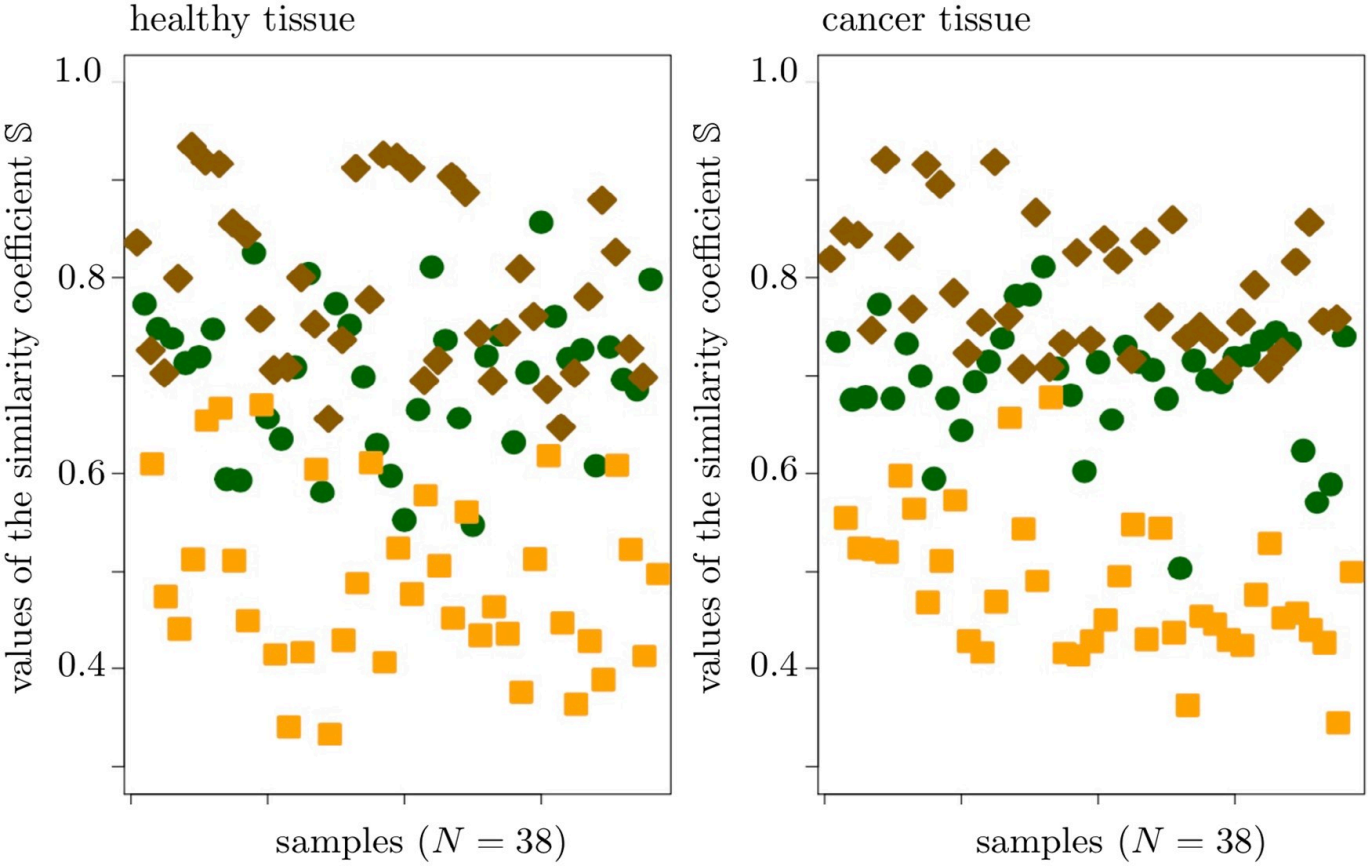
Pairwise similarity of the 5hmC measures Δ*β*(100), Δ*m*^∞^ and Δ*h*, in terms of the similarity coefficient S. Orange rectangles correspond to the values of S(Δβ(100),Δh), dark green dots to the values of $S(\Delta \beta \left(100\right),\Delta m^{\infty })$ and brown rectangles to the values of $S(\Delta h,\Delta m^{\infty })$.

While performing the similarity analysis for each given sample, we could not state any significant difference in the values of S as computed on healthy and cancer tissue. Further, the 5hmC measures Δ*m*^∞^ and Δ*h* appears to be the most similar, whereas the measures Δ*h* and Δ*β*(100) are the least similar, both on healthy and cancer tissue. Finally, the 5hmC measure Δ*β*(100) is less similar to Δ*h* than to Δ*m*^∞^, both on healthy and cancer tissue. All these results are visualized in Fig 9.

To describe the distribution of S for any two given 5hmC measures, we adapted the ideas of [56, 57] and calculated the expected value of this similarity coefficient. The results of those calculations are presented in the left-hand panel of Table 1. Due to that table, on cancer tissue the measures Δ*m*^∞^ and Δ*h* are again the most similar 5hmC measures; further, Δ*β*(100) and Δ*h* are the least similar to each other, both on healthy and cancer tissue.

**Table 1:** Expected values of the similarity coefficients S and $S_{H}$.

	E[S(.,.)]		$E[S_{H}\left(.,.\right)]$	
5hmC measures	healthy tissue	cancer tissue	healthy tissue	cancer tissue
Δ*β*(100), Δ*h*	0.7946	0.7897	0.5892	0.5794
Δ*β*(100), Δ*m*^∞^	0.8052	0.8016	0.6104	0.6032
Δ*m*^∞^, Δ*h*	0.8021	0.8060	0.6042	0.6120

Expected values of the similarity coefficients S and $S_{H}$ as applied for the pairwise comparison of the 5hmC measures Δ*β*(100), Δ*m*^∞^ and Δ*h*.

The similarity coefficient S in (13) exhibits a number of advantages such as simple applicability and intuitive interpretation of the obtained values. However, there are also some issues related to this coefficient. One of these issues arises in situations with two 5hmC measures *x*_1_ and *x*_2_ characterized by

$$\begin{array}{c} \sum_{i=1}^{n}I_{\left\{x_{1}^{i}>0\right\}}I_{\left\{x_{2}^{i}>0\right\}}=0. \end{array}$$ (14)

For such 5hmC measures, which should actually be considered as completely dissimilar in the context of 5hmC detection, there is still a real possibility to get a positive value of the coefficient S as

$$\begin{array}{c} S\left(x_{1},x_{2}\right)=\frac{1}{n}\sum_{i=1}^{n}I_{\left\{x_{1}^{i}\leq 0\right\}}I_{\left\{x_{2}^{i}\leq 0\right\}} \end{array}$$ (15)

which may indeed become misleading in the context of the screening step. This situation will even deteriorate for $\sum_{i=1}^{n}I_{\left\{x_{1}^{i}\leq 0\right\}}I_{\left\{x_{2}^{i}\leq 0\right\}}\to n.$

To mitigate this issue, we consider the *similarity coefficient of Hamann*, $S_{H},$ defined as

$$\begin{array}{c} \begin{array}{cc} S_{H}\left(x_{1},x_{2}\right) & =\frac{1}{n}(\sum_{i=1}^{n}I_{\left\{x_{1}^{i}>0\right\}}I_{\left\{x_{2}^{i}>0\right\}}+\sum_{i=1}^{n}I_{\left\{x_{1}^{i}\leq 0\right\}}I_{\left\{x_{2}^{i}\leq 0\right\}}\\ & -\sum_{i=1}^{n}I_{\left\{x_{1}^{i}>0\right\}}I_{\left\{x_{2}^{i}\leq 0\right\}}-\sum_{i=1}^{n}I_{\left\{x_{1}^{i}\leq 0\right\}}I_{\left\{x_{2}^{i}>0\right\}}). \end{array} \end{array}$$ (16)

Clearly, $S_{H}$ is just a transformation of the simple matching coefficient S [53] that incorporates a correction for possible mismatches between the considered 5hmC measures *x*_1_ and *x*_2_. While ranging in the interval [−1, 1], $S_{H}\left(x_{1},x_{2}\right)=-1$ can be interpreted as *complete dissimilarity* and $S_{H}\left(x_{1},x_{2}\right)=1$ as *complete similarity* between *x*_1_ and *x*_2_. Further, due to (13) and (16), $S_{H}\left(x_{1},x_{2}\right)\leq S\left(x_{1},x_{2}\right)$ for any two measures *x*_1_ and *x*_2_.

As in case with S, we calculated the expected value of $S_{H}$ for any two given 5hmC measures; the results are presented in the right-hand panel of Table 1. As expected, this table shows the similarity coefficient $S_{H}$ confirming the results obtained under S, e.g., with the 5hmC measures Δ*m*^∞^ and Δ*h* being most similar to each other on cancer tissue.

Altogether, we state that among three considered 5hmC measures Δ*β*(*α*), Δ*m*^∞^ and Δ*h*, the measures Δ*m*^∞^ and Δ*h* appear to be most similar to each other on cancer tissue, both in terms of S and $S_{H}$. Further, as in case of the prevalence of positive results analysis, the measure Δ*m*^∞^ is more similar to Δ*β*(*α*) than the measure Δ*h* is.

### Relative accuracy analyses

A different aproach for addressing the pairwise resemblance of the proposed 5hmC measures is to consider their *relative* sensitivities *SE*_*r*_, specificities *SP*_*r*_ and false discovery rates *FDR*_*r*_. Here, for any two 5hmC measures *x*_1_ and *x*_2_, we set $SE_{r}\left(x_{1}\;|\;x_{2}\right)=P\left(x_{1}>0\;|\;x_{2}>0\right)$ as the relative sensitivity of *x*_1_ with respect to *x*_2_, $SP_{r}\left(x_{1}\;|\;x_{2}\right)=P\left(x_{1}\leq 0\;|\;x_{2}\leq 0\right)$ as the relative specificity of *x*_1_ with respect to *x*_2_ and

$$\begin{array}{c} FDR_{r}\left(x_{1}\;|\;x_{2}\right)=1-\frac{P(x_{1}>0,x_{2}>0)}{P(x_{1}>0)}=1-SE_{r}\left(x_{2}\;|\;x_{1}\right) \end{array}$$ (17)

as the relative false discovery rate. The quantities *SE*_*r*_ and *SP*_*r*_ are also known as *co-positivities* and *co-negativities*, respectively [58, 59].

We started our data analyses on relative accuracies by checking for a significant difference in relative sensitivities as computed on healthy and cancer tissue. In a sample-wise analysis, such difference was observed for the relative sensitivities *SE*_*r*_(Δ*β*(100)|Δ*h*) and *SE*_*r*_(Δ*β*(100)|Δ*m*^∞^), with the relative sensitivity on healthy tissue exceeding the corresponding relative sensitivity on cancer tissue. The same analysis, performed CpG-wise, showed all relative sensitivities differentiating significantly between healthy and cancer tissue.

Further, in a sample-wise analysis, performed on healthy tissue, the 5hmC measure Δ*m*^∞^ demonstrated a higher sensitivity with respect to Δ*h* than Δ*h* did with respect to Δ*m*^∞^. This is consistent with our results on prevalence of positive results, with the measure Δ*m*^∞^ being less conservative than Δ*h* on healthy tissue. Further, there was a trend for a significant increase in the relative sensitivity *SE*_*r*_(Δ*m*^∞^|Δ*β*(100)) compared to the relative sensitivity *SE*_*r*_(Δ*β*(100)|Δ*m*^∞^) on cancer tissue. This is also related to our result on prevalence of positive results, with the measure Δ*m*^∞^ being less conservative than Δ*β*(100) on cancer tissue.

A CpG-wise analysis of relative sensitivities revealed, the 5hmC measure Δ*β*(100) showing a lower sensitivity with respect to the measure Δ*h* than the other way around, on cancer tissue. This result changed to the opposite on healthy tissue. Further, the measure Δ*m*^∞^ showed a higher sensitivity with respect to the measure Δ*β*(100) than Δ*β*(100) did with respect to Δ*m*^∞^, both on healthy and cancer tissue. Analogous result was true for the measures Δ*m*^∞^ and Δ*h*, with *SE*_*r*_(Δ*m*^∞^|Δ*h*) exceeding *SE*_*r*_(Δ*h*|Δ*m*^∞^), both on healthy and cancer tissue.

While analyzed sample-wise for its relative specificity, the measure Δ*β*(100) demonstrated a significantly lower specificity with respect to Δ*h* on healthy tissue than on cancer tissue; similar result holds for relative specificity of the measure Δ*m*^∞^ with respect to the measure Δ*h*. The same analysis, performed CpG-wise, showed all relative specificities differentiating significantly between healthy and cancer tissue. Further, the 5hmC measure Δ*β*(100) demonstrated a higher specificity with respect to the measure Δ*m*^∞^ than Δ*m*^∞^ did with respect to Δ*β*(100), both on healthy and cancer tissue, with the difference being more substantial on cancer tissue. This is again in correspondence with the measure Δ*m*^∞^ being less conservative than Δ*β*(100), in particular on cancer tissue.

In a CpG-wise analysis, on healthy tissue the measure Δ*β*(100) demonstrated a significantly lower specificity with respect to the measure Δ*h* than Δ*h* did with respect to Δ*β*(100); this result changes to the opposite while considering the same relative specificities on cancer tissue. Further, the measure Δ*m*^∞^ showed a lower specificity with respect to Δ*β*(100) than Δ*β*(100) did with respect to Δ*m*^∞^, on both considered tissues; similar result is true for the measures Δ*m*^∞^ and Δ*h*.

Due to its definition, the results on relative false discovery rates *FDR*_*r*_ can be immediately derived from the corresponding results on *SE*_*r*_. For instance, one can show that the measure Δ*h* has a higher false discovery rate with respect to Δ*β*(100) than Δ*m*^∞^, both on healthy and cancer tissue.

We also computed expected relative sensitivities, specificities and false discovery rates of each 5hmC measure with respect to two others; the results are presented in Tables 2–4 below.

**Table 2:** Expected relative sensitivities $E[SE_{r}\left(x_{1}\;|\;x_{2}\right)]$.

	healthy tissue			cancer tissue		
$E[SE_{r}]$	Δ*m*^∞^	Δ*β*(100)	Δ*h*	Δ*m*^∞^	Δ*β*(100)	Δ*h*
Δ*m*^∞^	1.0	0.7739	0.8624	1.0	0.7827	0.8383
Δ*β*(100)	0.7456	1.0	0.5938	0.7133	1.0	0.5549
Δ*h*	0.7940	0.5613	1.0	0.8199	0.5906	1.0

Expected relative sensitivities $E[SE_{r}\left(x_{1}\;|\;x_{2}\right)]$, computed for any two 5hmC measures *x*_1_, *x*_2_ ∈ {Δ*β*(100), Δ*m*^∞^, Δ*h*}. The measure *x*_1_ is in rows and *x*_2_ is in columns; e.g., the value 0.7456 corresponds to the expected relative sensitivity $E[SE_{r}\left(\Delta \beta \left(100\right)\;|\;\Delta m^{\infty }\right)]$ and the value 0.7739 to the expected relative sensitivity $E[SE_{r}\left(\Delta m^{\infty }\;|\;\Delta \beta \left(100\right)\right)]$, both on healthy tissue. Since larger values in the table describe a higher relative sensitivity, our results in the table above indicate the measures Δ*h* and Δ*m*^∞^ demonstrating the highest resemblance. At the same time, the measures Δ*β*(100) and Δ*h* appear to be least similar to each other.

**Table 3:** Expected relative specificities $E[SP_{r}\left(x_{1}\;|\;x_{2}\right)]$.

	healthy tissue			cancer tissue		
$E[SP_{r}]$	Δ*m*^∞^	Δ*β*(100)	Δ*h*	Δ*m*^∞^	Δ*β*(100)	Δ*h*
Δ*m*^∞^	1.0	0.5698	0.6844	1.0	0.5669	0.7112
Δ*β*(100)	0.5982	1.0	0.3473	0.6455	1.0	0.3834
Δ*h*	0.7890	0.3788	1.0	0.7422	0.3529	1.0

Expected relative specificities $E[SP_{r}\left(x_{1}\;|\;x_{2}\right)]$, computed for any two 5hmC measures *x*_1_, *x*_2_ ∈ {Δ*β*(100), Δ*m*^∞^, Δ*h*}. The measure *x*_1_ is in rows and *x*_2_ is in columns; e.g., the value 0.5982 corresponds to the expected relative specificity $E[SP_{r}\left(\Delta \beta \left(100\right)\;|\;\Delta m^{\infty }\right)]$ and the value 0.5698 to the expected relative specificity $E[SP_{r}\left(\Delta m^{\infty }\;|\;\Delta \beta \left(100\right)\right)]$, both on healthy tissue. As in Table 2, larger values correspond to a higher relative specificity, and thus Δ*β*(100) and Δ*m*^∞^ demonstrate a higher resemblance than Δ*β*(100) and Δ*h*.

**Table 4:** Expected relative false discovery rates $E[FDR_{r}\left(x_{1}\;|\;x_{2}\right)]$.

	healthy tissue			cancer tissue		
$E[FDR_{r}]$	Δ*m*^∞^	Δ*β*(100)	Δ*h*	Δ*m*^∞^	Δ*β*(100)	Δ*h*
Δ*m*^∞^	0.0	0.2544	0.2060	0.0	0.2867	0.1801
Δ*β*(100)	0.2261	0.0	0.4387	0.2173	0.0	0.4094
Δ*h*	0.1376	0.4062	0.0	0.1617	0.4451	0.0

Expected relative false discovery rate $E[FDR_{r}\left(x_{1}\;|\;x_{2}\right)]$, computed for any two 5hmC measures *x*_1_, *x*_2_ ∈ {Δ*β*(100), Δ*m*^∞^, Δ*h*}. The measure *x*_1_ is in rows and *x*_2_ is in columns; e.g., the value 0.2261 corresponds to the expected relative false discovery rate $E[FDR_{r}\left(\Delta \beta \left(100\right)\;|\;\Delta m^{\infty }\right)]$ and the value 0.2544 to $E[FDR_{r}\left(\Delta m^{\infty }\;|\;\Delta \beta \left(100\right)\right)]$, both on healthy tissue. Altogether, the measure Δ*m*^∞^ demonstrates a lower false discovery rate with respect to Δ*β*(100) than Δ*h*.

Altogether, due to our relative accuracy analyses, the measure Δ*m*^∞^ again demonstrates more resemblance with Δ*β*(100) than the measure Δ*h*, both on healthy and cancer tissue.

### Comparison of Δ*β*(*α*), Δ*h* and Δ*m*^∞^ to the oxBS-MLE and OxyBS procedures in the context of a screening step

When detecting CpGs with a substantial 5hmC level, one may compare the results provided by each of the considered three 5hmC measures Δ*β*(*α*), Δ*h* and Δ*m*^∞^ with those derived from the oxBS-MLE and OxyBS procedures introduced in [44, 47]. When applied in a screening step, both oxBS-MLE and OxyBS procedures will flag the same cytosines as being hydroxymethylated as the 5hmC measure Δ*β*(0) will do. This results follows immediately from the problem formulations and the derivation of the MLEs as suggested by both procedures; see S4 Appendix for details. Thus, the comparison of the 5hmC measures Δ*m*^∞^ and Δ*h* with the oxBS-MLE and OxyBS procedures in detection of hydroxymethylated cytosines can be traced back to the comparison of these measures with the measure Δ*β*(0).

## Conclusion

Presently, the measure most commonly used for the detection of hydroxymethylated CpGs is the measure Δ*β*(*α*) and its derivatives as introduced in [31, 41, 42]. Well-established due to its easy computation and alleged intuitivity, this 5hmC measure nevertheless exhibits a number of limitations and has already been criticized due to its interpretation. This interpretation has meanwhile been questioned in [44], where the authors discussed the “naive” estimation of the 5hmC level via the difference of two *β* values as proposed in [31, 41] and introduced a model for describing the 5mC and 5hmC proportions by means of maximum likelihood estimation and beta-distributed random variables. Such modeling disallows negative proportions in particular; the corresponding model was also implemented in the *R*-package *OxyBS* [44].

In this paper, we performed a detailed analysis of Δ*β*(*α*), both analytically and empirically, and discussed a number of limitations of Δ*β*(*α*) which could make its practical applicability for screening of hydroxymethylated CpGs questionable. These limitations concern in particular the interpretation of Δ*β*(*α*) and its robustness with respect to the choice of *α*.

Further, we proposed two alternative 5hmC measures which can be applied in the screening step. The first of these 5hmC measures is the measure Δ*m*^∞^. While intuitively interpretable and independent of the correction term *α*, this measure does not incorporates the unmethylated intensities *U*_*BS*_ and *U*_*oxBS*_. Even though the role of these intensities in detection of the 5hmC levels has not been clarified yet, we took this fact into account and suggested the second alternative 5hmC measure, Δ*h*. Due to its definition, this measure does not depend on the choice of *α*, has an intuitive interpretation in detecting hydroxymethylated CpGs, takes into account all intensities, and can be computed directly from the observed data.

The main challenge to be handled in our analysis referred to a mutual comparison of the considered 5hmC measures in the *absence of a gold standard*, as no biological or biochemical criterion for a CpG to be considered as “hydroxymethylated”, e.g., in terms of methylated and non-methylated intensities *M* and *U*, is available so far. To overcome this challenge and to be able to address resemblance of the proposed 5hmC measures in the context of the screening step, we first analyzed the prevalences of positive results for each single 5hmC measure. Here, we first observed a decrease in this prevalence, while moving from healthy to cancer tissue, for the measures Δ*β*(*α*) and Δ*m*^∞^. This result is also in accordance with the observation on a depletion of 5hmC levels in tumors compared to corresponding normal tissue as stated, e.g., in [24, 45, 60]. Moreover, the measure Δ*m*^∞^ appears to be the measure with the largest prevalence of positive results, both on healthy and cancer tissue. In addition, data-based analysis of the joint prevalence of positive results revealed the strongest agreement between the measures Δ*m*^∞^ and Δ*h*, followed by the agreement between the measures Δ*β*(100) and Δ*m*^∞^; the 5hmC measures Δ*β*(100) and Δ*h* demonstrated the lowest pairwise agreement. In other words, a stronger resemblance between the measures Δ*β*(*α*) and Δ*m*^∞^ than between the measures Δ*β*(*α*) and Δ*h* was observed so far. This result was also confirmed in the context of a similarity analysis as performed for a pairwise comparison of the proposed 5hmC measures.

In order to estimate relative accuracies of Δ*β*(100), Δ*m*^∞^, and Δ*h* with respect to each other, we also used relative sensitivity and specificity analyses. As a result of those analyses, the measure Δ*m*^∞^ demonstrated a higher sensitivity and a lower specificity with respect to Δ*β*(100) than vice versa; the same result holds for the measures Δ*m*^∞^ and Δ*h*. Moreover, we observed that the measure Δ*h* has a higher false discovery rate with respect to Δ*β*(100) than Δ*m*^∞^. Altogether, we concluded, that, in the context of the screening step, the 5hmC measure Δ*m*^∞^ exhibits more resemblance with the measure Δ*β*(*α*) than Δ*h* does and thus this measure would be the first choice if looking for a possible substitute for Δ*β*(*α*) with another 5hmC measure in the screening procedure.

Our numerical analyses are based on raw data, with no normalization method applied. There are a variety of reasons for this. First, some of our results (such as the convergence result for Δ*β*(*α*)) were derived analytically and thus do not depend on the data used for their illustration. Second, there is no consistent normalization method to be applied when quantifying the 5hmC levels [42, 44]. Third, a possible impact of a particular normalization method on the results of the 5hmC classification is currently not obvious to us and can in fact be considered as a topic of future research.

Nevertheless, we did check our results on the data normalized by three different normalization methods, *funNorm*, *SWAN* and *Illumina*, as available in the *R*-package *minfi* [51]; for more details see S5 Appendix. As a consequence of such normalized data analyses, we do observe some differences to our results as obtained on raw data. However, there is no evidence that such differences have any biological meaning and are not just a product of the normalization method applied. For instance, in some cases we observe a reduction in the prevalence of positive results of a given 5hmC measure as calculated on normalized data compared to raw data. On the other hand, a reduction in the 5hmC levels on cancer tissue as observed in terms of the measure Δ*β*(100) is confirmed for all three normalized data sets as well. The same is true for the measure Δ*m*^∞^ being less conservative than Δ*h* on healthy tissue. Further, the measures Δ*m*^∞^ and Δ*h* are the ones that are most similar to each other (in terms of the similarity coefficient S) followed by the measures Δ*β*(100) and Δ*m*^∞^, both on raw and normalized data; this result holds both for healthy and cancer tissue.

There are also differences in results on detection of the hydroxymethylated CpGs provided by different normalization procedures. For instance, on cancer tissue, the measure Δ*β*(100) shows a significant reduction in the prevalence of positive results calculated on the *Illumina* data compared to the prevalence computed on the *funNorm* data. Further, both on healthy and cancer tissue, the measures Δ*β*(100) and Δ*m*^∞^ demonstrate the strongest similarity (in terms of the similarity coefficient S) on the *funNorm* normalized data, followed by the *SWAN* normalized data; the similarity between Δ*β*(100) and Δ*m*^∞^ on the *Illumina* normalized data is the lowest one.

In the present paper we discussed the possible applicability of the considered 5hmC measures for detection of hydroxymethylated CpGs in the screening procedure. The immediate question arising in this context is the question about the applicability of these measures for the quantification of the observed 5hmC levels, similar to the applicability of *β* values used for quantification of the methylation levels. Even if the measure Δ*h* appears to provide the most intuitive interpretation in contrast to the remaining two 5hmC measures, this question is still a topic of future research.

## Supporting information

S1 Appendix On the 5hmC measure Δ*β*(*α*). Sign change of Δ*β*(*α*), sample-wise convergence of the CpG sets satisfying {Δ*β*(*α*) > 0} as *α* increases, the role of *α* in similarity analyses.
(PDF)

S2 Appendix On the 5hmC measures Δ*m*(*α*). Relation between the measures Δ*m*(*α*) and Δ*β*(*α*), the 5hmC measure Δ*m*(*α*) as a function of *α* (monotonicity, convergence, sign change of Δ*m*(*α*)), relation between the subsets {Δ*m*(*α*) > 0} and {Δ*m*^∞^ > 0}, relation between the subsets {Δ*h* > 0}, {Δ*β*(*α*) > 0} and {Δ*m*^∞^ > 0}.
(PDF)

S3 Appendix On the resemblance of Δ*β*(*α*), Δ*m*^∞^ and Δ*h*: Numerical results. Prevalence of positive results, joint prevalence of positive results, similarity analyses, relative accuracy analyses (relative sensitivity and specificity).
(PDF)

S4 Appendix A comparison of the 5hmC measures Δ*β*(*α*), Δ*h* and Δ*m*^∞^ with the results of the oxBS-MLE and OxyBS procedures. (PDF)

S5 Appendix A comparison of numerical analyses on raw and normalized data. (PDF)
