# Supplementary material for: Statistical methods for classification of 5hmC levels based on the Illumina Inifinium HumanMethylation450 (450k) array data, under the paired bisulfite (BS) and oxidative bisulfite (oxBS) treatment
Source: PLoS One. 2019 Jun 13;14(6):e0218103. doi: 10.1371/journal.pone.0218103 (PMC6563990; doi:10.1371/journal.pone.0218103)
Supplement: S1 Appendix — Sign change of Δβ(α), sample-wise convergence of the CpG sets satisfying {Δβ(α) > 0} as α increases, the role of α in similarity analyses. (PDF) [file pone.0218103.s001.pdf]

Statistical methods for classification of 5hmC levels based on the Illumina Inifinium HumanMethylation450 (450k) array data, under the paired bisulfite (BS) and oxidative bisulfite (oxBS) treatment.

S1 Appendix: On the 5hmC measure $\Delta\beta(\alpha)$

Alla Slynko[1], Axel Benner[2] June 1, 2019

Sign change of $\Delta\beta(\alpha)$

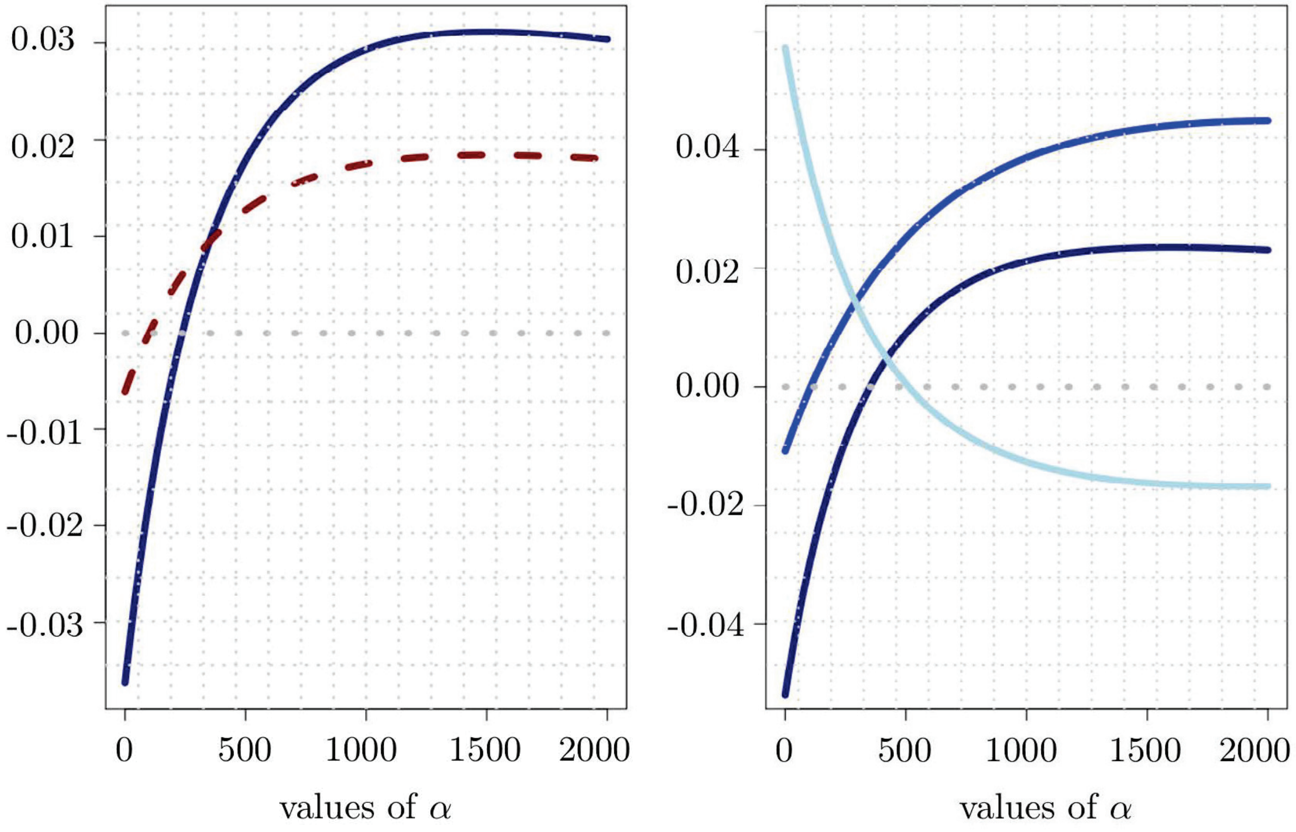

Fig A. Sign change of the 5hmC measure $\Delta\beta(\alpha)$. The left-hand panel: the sign change both for healthy (at $\alpha = 237.36$, the dark blue curve) and cancer (at $\alpha = 100$, the dark red curve) tissue, sample 2 and CpG *cg*01520478. The right-hand panel: the sign change for CpGs cg07643829 (at $\alpha = 105.48$, the blue curve), cg03604216 (at $\alpha = 351.49$, the dark blue curve) and cg05861697 (at $\alpha = 511.36$, the light blue curve), healthy tissue and sample 2.

[1]Department of Statistics and Actuarial Science, University of Waterloo, Waterloo, Canada, alla.a.slynko@gmail.com
[2]Division of Biostatistics, German Cancer Research Center, Heidelberg, Germany

Samplewise convergence of the CpG sets satisfying $\{\Delta\beta(\alpha) > 0\}$ as α increases

For a given sample, the percentage of CpGs satisfying the condition $\Delta\beta(\alpha) > 0$ approaches a positive constant as α increases. Standard computations, and the relation between the measures $\Delta\beta(\alpha)$ and $\Delta m(\alpha)$, as described in S2 Appendix, verify this limit value to be just the percentage of CpGs satisfying $M_{BS} > M_{oxBS}$ for the corresponding sample; see Fig B for an illustration of this result. Note that the considered limit value is actually given by the percentage of CpGs satisfying

$$\{M_{BS} > M_{oxBS}\} \cup \{M_{BS} = M_{oxBS}, U_{BS} < U_{oxBS}\},$$

but for simplicity we ignore the latter set, in particular, due to its evident irrelevance for the quantification of 5hmC level.

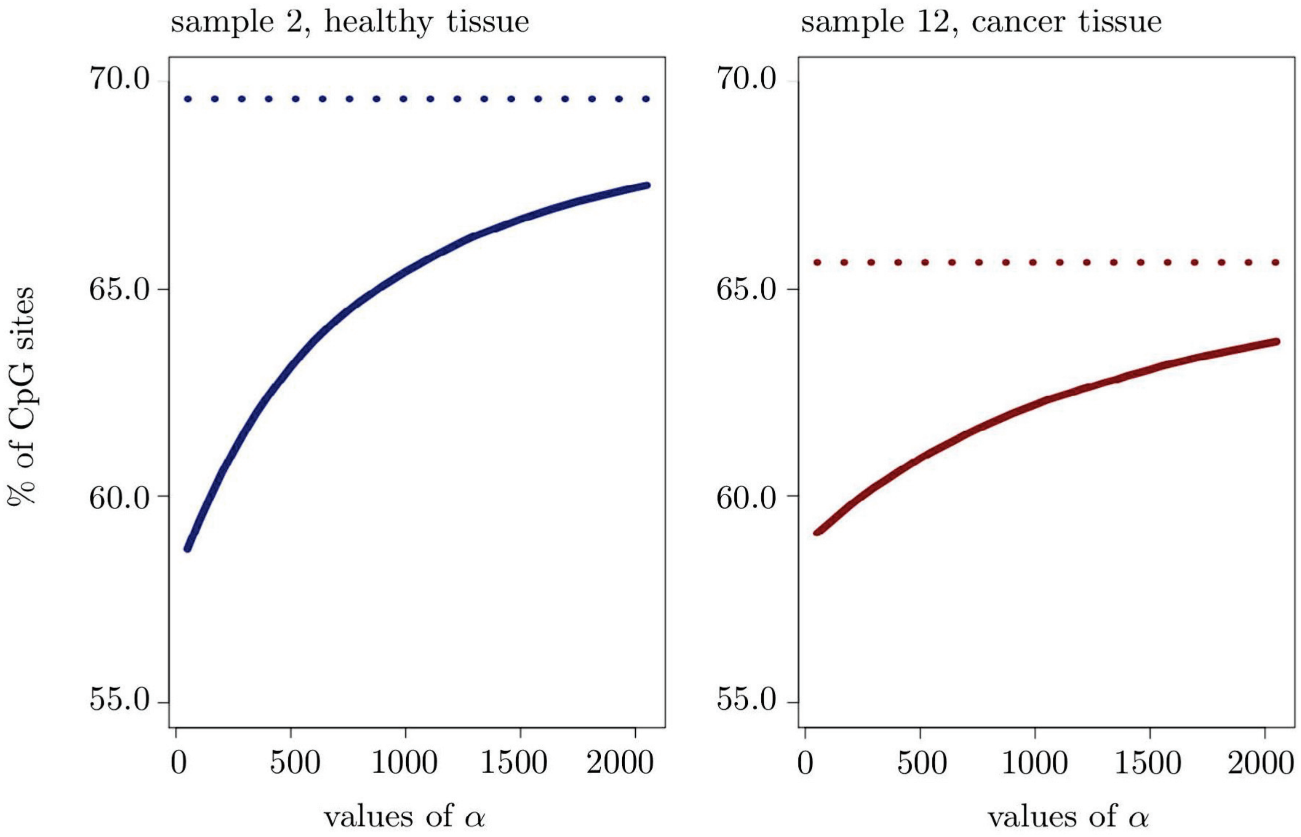

Fig B. The percentage of CpGs satisfying $\Delta\beta(\alpha) > 0$ as α increases. A real data example computed for two given samples and healthy (the left-hand panel) as well as cancer (the right-hand panel) tissue. The dark blue horizontal line corresponds to the percentage of CpG sites satisfying $M_{BS} > M_{oxBS}$ for healthy tissue; the dark red horizontal line shows the analogous percentage for cancer tissue

Fig C demonstrates an increase in the joint prevalence of positive results between $\Delta\beta(\alpha)$ and each of both remaining 5hmC measures as α grows.

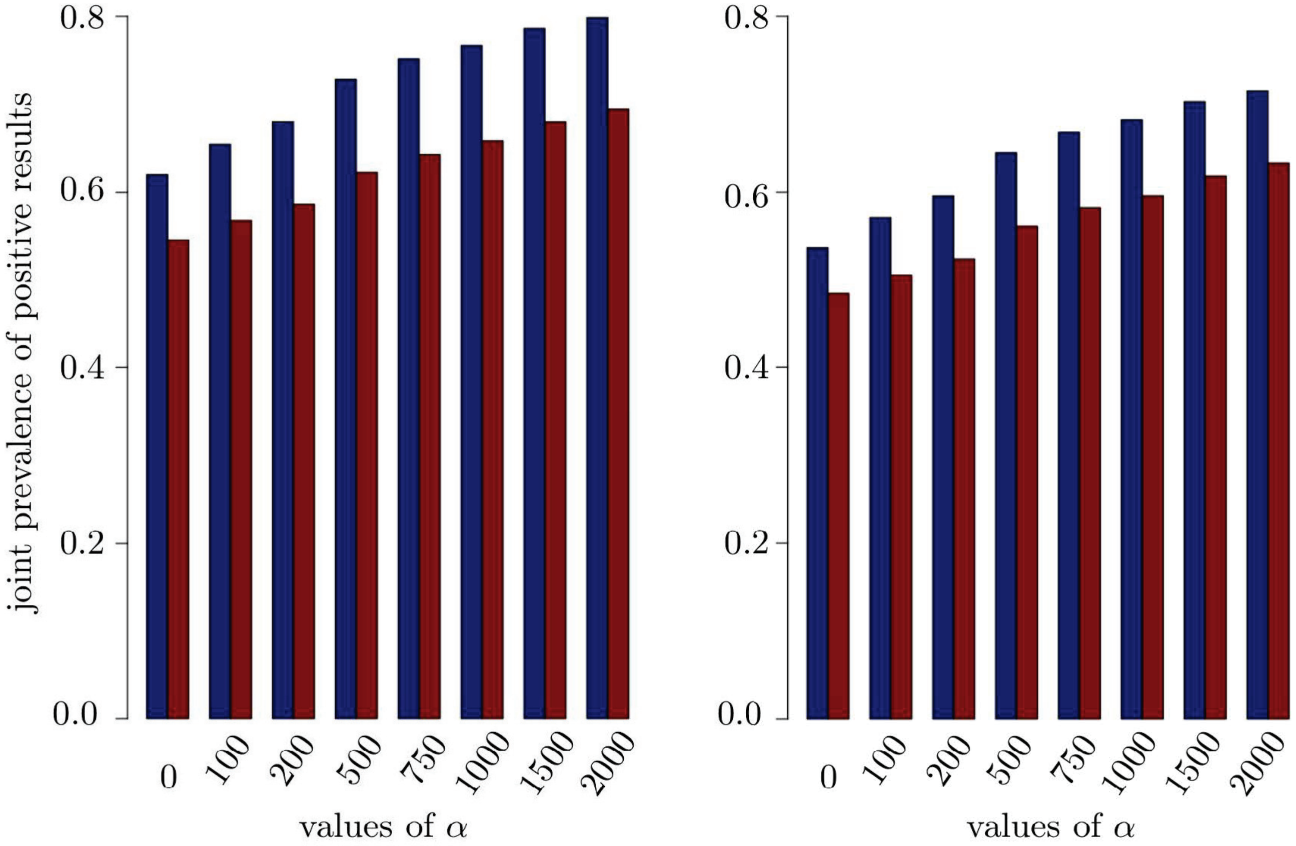

Fig C. Joint prevalence of positive results: dependence on α. The left-hand panel: The percentage of CpGs satisfying simultaneously the conditions $\Delta\beta(\alpha) > 0$ and $\Delta m^{\infty} > 0$, for increasing α. The right-hand panel: The percentage of CpGs satisfying simultaneously the conditions $\Delta\beta(\alpha) > 0$ and $\Delta h > 0$, for increasing α. All calculations were performed for a given sample (*sample 1*). The blue bars correspond to healthy tissue, the red bars to cancer tissue.

The role of α in similarity analyses

Due to Fig B, the percentage of CpGs satisfying $\Delta\beta(\alpha) > 0$ for a given sample grows with increasing values of α. While addressing this observation in the context of pairwise resemblance of $\Delta\beta(\alpha)$ to each of both other 5hmC measures, Fig D demonstrates an increasing pairwise similarity between $\Delta\beta(\alpha)$ and each of the considered 5hmC measures Δm^{∞} and Δh as α increases.

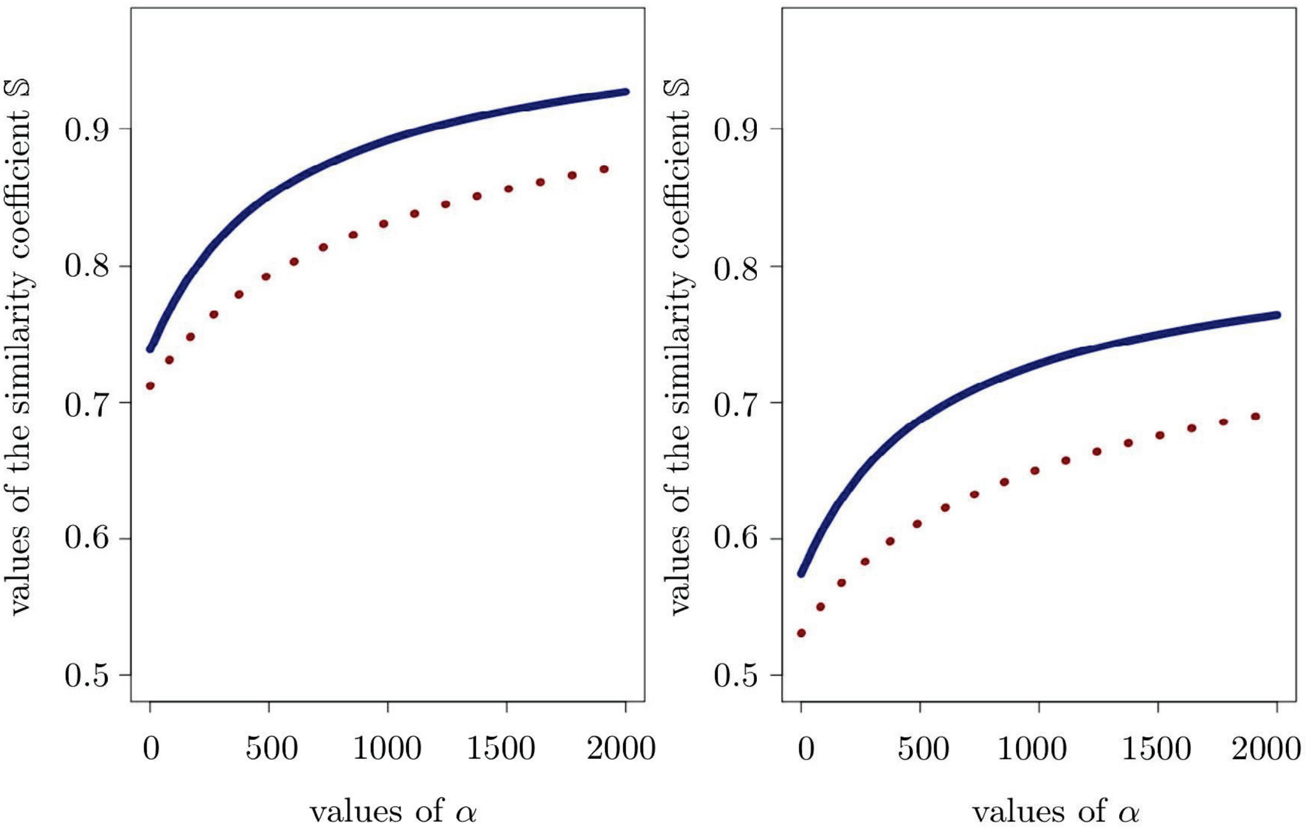

Fig D. Pairwise similarity for the measures $\boldsymbol{\Delta\beta(\alpha)}$, $\boldsymbol{\Delta m^{\infty}}$ and $\boldsymbol{\Delta h}$ and increasing values of $\boldsymbol{\alpha}$. The left-hand panel: Similarity between the measures $\Delta\beta(\alpha)$ and Δm^{∞}. The right-hand panel: Similarity between the measures $\Delta\beta(\alpha)$ and Δh. All calculations were performed for a given sample and in terms of the similarity coefficient $\mathbb{S}$. The dark blue lines correspond to healthy tissue, the dark red dotted lines to cancer tissue.
