# Supplementary material for: Statistical methods for classification of 5hmC levels based on the Illumina Inifinium HumanMethylation450 (450k) array data, under the paired bisulfite (BS) and oxidative bisulfite (oxBS) treatment
Source: PLoS One. 2019 Jun 13;14(6):e0218103. doi: 10.1371/journal.pone.0218103 (PMC6563990; doi:10.1371/journal.pone.0218103)
Supplement: S2 Appendix — Relation between the measures Δm(α) and Δβ(α), the 5hmC measure Δm(α) as a function of α (monotonicity, convergence, sign change of Δm(α)), relation between the subsets {Δm(α) > 0} and {Δm∞ > 0}, relation between the subsets {Δh > 0}, {Δβ(α) > 0} and {Δm∞ > 0}. (PDF) [file pone.0218103.s002.pdf]

Statistical methods for classification of 5hmC levels based on the Illumina Inifinium HumanMethylation450 (450k) array data, under the paired bisulfite (BS) and oxidative bisulfite (oxBS) treatment.

S2 Appendix: On the 5hmC measure $\Delta m(\alpha)$

Alla Slynko[1], Axel Benner[2] June 1, 2019

On the 5hmC measure $\Delta m(\alpha)$

Relation between the measures $\Delta m(\alpha)$ and $\Delta\beta(\alpha)$

By adopting the idea of $\Delta\beta$ defined as a difference of two respective β values, the authors in [42] consider the measure

$$\Delta m = m_{BS} - m_{oxBS} = \log_2 \frac{\beta_{BS}}{1-\beta_{BS}} - \log_2 \frac{\beta_{oxBS}}{1-\beta_{oxBS}} \tag{1}$$

as a difference of two respective m-values [13] and a possible alternative to the measure $\Delta\beta$. Note that due to [13], any m-value is just a transformation of the corresponding β value, namely

$$m = \log_2 \frac{\beta}{1-\beta}. \tag{2}$$

In contrast, there is no formal transformation between Δm and $\Delta\beta$ that would render Δm as a function of $\Delta\beta$.

We use standard calculations to transform (1) into

$$\Delta m(\alpha) = \log_2 \frac{M_{BS}}{M_{oxBS}} + \log_2 \frac{U_{oxBS}+\alpha}{U_{BS}+\alpha}. \tag{3}$$

With (3), the condition $\Delta m(\alpha) > 0$ is equivalent to the inequality

$$\frac{M_{BS}}{M_{oxBS}} > \frac{U_{BS}+\alpha}{U_{oxBS}+\alpha}. \tag{4}$$

This inequality also leads to $\Delta\beta(\alpha) > 0$. Thus, for any given intensities M_{BS}, M_{oxBS}, U_{BS}, U_{oxBS} and $\alpha > 0$ the measures $\Delta m(\alpha)$ and $\Delta\beta(\alpha)$ must have the same sign. Indeed, for any $\alpha > 0$, the condition $\Delta m(\alpha) > 0$ implies

$$\frac{\beta_{BS}(\alpha)}{\beta_{oxBS}(\alpha)} \cdot \frac{1-\beta_{oxBS}(\alpha)}{1-\beta_{BS}(\alpha)} > 1$$

[1]Department of Statistics and Actuarial Science, University of Waterloo, Waterloo, Canada, alla.a.slynko@gmail.com
[2]Division of Biostatistics, German Cancer Research Center, Heidelberg, Germany

which is equivalent to $\beta_{BS}(\alpha) > \beta_{oxBS}(\alpha)$ or, alternatively, $\Delta\beta(\alpha) > 0$. On the other hand, the condition $\Delta\beta(\alpha) > 0$ leads to the inequality (4) and thus $\Delta m(\alpha) > 0$ follows.

Since the condition $\Delta m(\alpha) > 0$ holds in the same cases as the condition $\Delta\beta(\alpha) > 0$ does, at the end of the screening step, the two 5hmC measures $\Delta m(\alpha)$ and $\Delta\beta(\alpha)$ will flag the same CpGs as being hydroxymethylated, for any given value of $\alpha > 0$.

In this context, these two measures can be applied *interchangeably* when detecting CpGs with a substantial level of hydroxymethylation.

As a matter of fact, for any given sample, CpG and positive value of α, two measures $\Delta\beta(\alpha)$ and $\Delta m(\alpha)$ will always have the same sign. This result, while contributing to the comparability of these two 5hmC measures as well as to the results in [41, 42], will at the same time lead to similar limitations in applicability of the measure $\Delta m(\alpha)$ while detecting hydroxymethylated CpGs as those of $\Delta\beta(\alpha)$. In particular, the measure $\Delta m(\alpha)$ exhibits the same ambiguities in the interpretation of its values as $\Delta\beta(\alpha)$ does; for instance, it is not evident how the conditions $\Delta m(\alpha) > 0$, $\Delta m(\alpha) = 0$ and $\Delta m(\alpha) < 0$ can be interpreted in terms of the 5hmC level observed for a given CpG and sample.

The 5hmC measure $\Delta m(\alpha)$ as a function of α

Monotonicity

We consider $\Delta m(\alpha)$ as a function of α and compute the derivative

$$\frac{d\Delta m(\alpha)}{d\alpha} = \frac{U_{BS} - U_{oxBS}}{(U_{BS} + \alpha)(U_{oxBS} + \alpha)\ln 2}. \tag{5}$$

This derivative is obviously non-negative for all CpGs with $U_{BS} \geq U_{oxBS}$. Thus, for such CpGs $\Delta m(\alpha)$ will increase with increasing α. On the other hand, for all CpGs with $U_{BS} < U_{oxBS}$ the measure $\Delta m(\alpha)$ is decreasing in α.

Since the derivative in (5) does not have zeros and, on the other hand, $\alpha \geq 0$ must hold, we set the minimum of $\Delta m(\alpha)$ to be attained at $\alpha = 0$ for all CpGs with $U_{BS} \geq U_{oxBS}$; the increasing values of α in this case will lead to increasing values of $\Delta m(\alpha)$. Similarly, for the CpGs with $U_{BS} < U_{oxBS}$, the maximal value of $\Delta m(\alpha)$ will be attained at $\alpha = 0$; the increasing values of α in this case will correspond to decreasing values of $\Delta m(\alpha)$.

Convergence

As already mentioned earlier, $\lim_{\alpha\uparrow\infty} \Delta\beta(\alpha) = 0$. Similarly, the convergence of $\Delta m(\alpha)$ for increasing α can be easily derived from (3), with

$$\lim_{\alpha\uparrow\infty} \Delta m(\alpha) = \log_2 \frac{M_{BS}}{M_{oxBS}}. \tag{6}$$

This limit also helped us to come to the definition of the 5hmC measure Δm^{∞} later.

Sign change of $\Delta m(\alpha)$

The values of $\Delta m(\alpha)$ depend on the choice of the correction term α, just as in case of the measure $\Delta\beta(\alpha)$; that is, $\Delta m(\alpha)$ may change its sign from positive to negative and vice versa under the same conditions the measure $\Delta\beta(\alpha)$ does. This issue is illustrated in Fig A. Note that such ability of $\Delta m(\alpha)$ to change its sign can produce misleading results in the context of the screening step, if this measure is used as the criterion for selecting CpGs with a substantial level of hydroxymethylation.

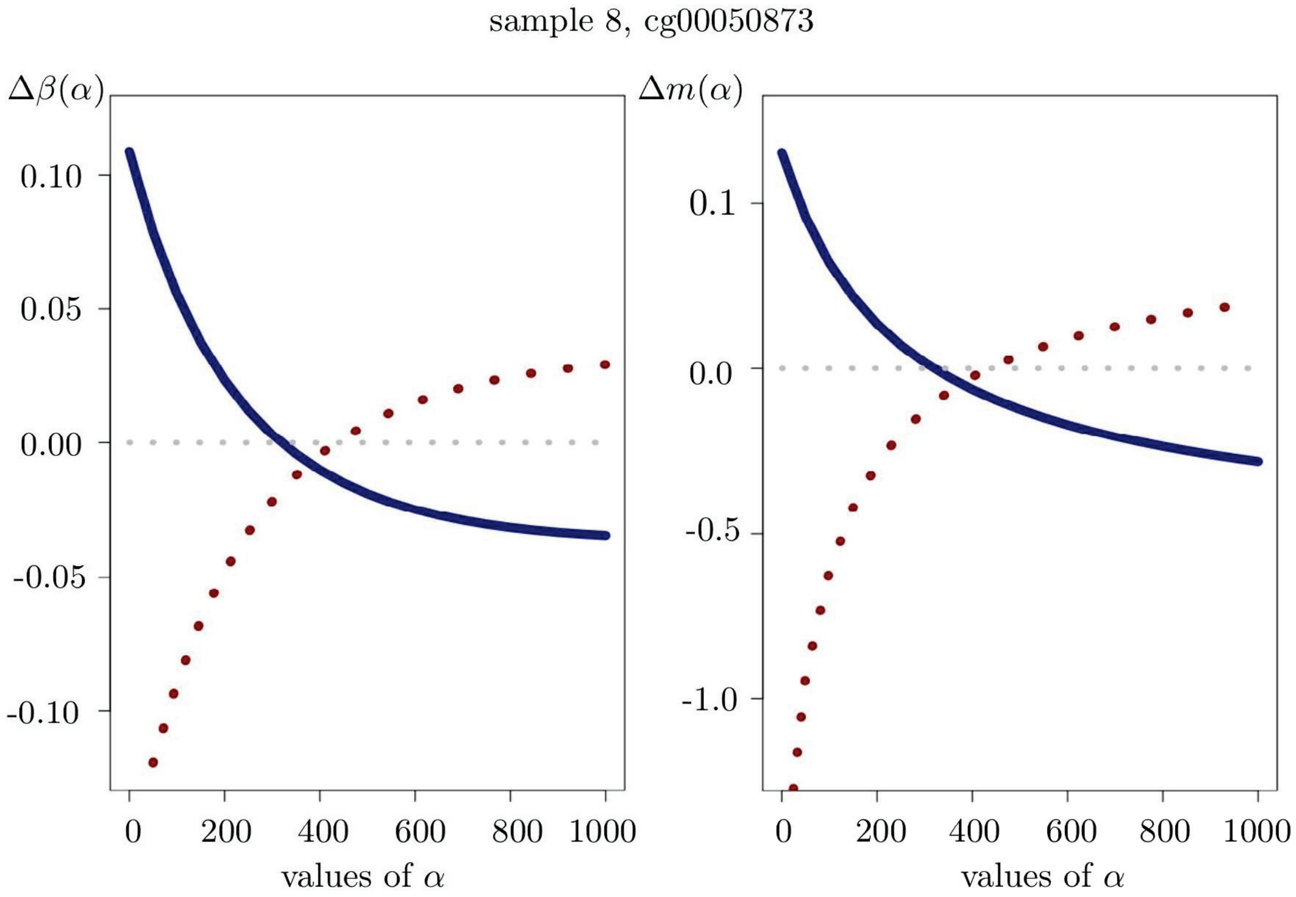

Fig A. Sign change of the measures $\Delta\beta(\alpha)$ and $\Delta m(\alpha)$. The measures $\Delta\beta(\alpha)$ (the left-hand panel) and $\Delta m(\alpha)$ (the right-hand panel) changing their signs as α increases, both on healthy (the blue curves) and cancer (the red curves) tissue. Note that both measures change their signs at the same values of $\alpha_1 = 320.3237$ for healthy tissue and $\alpha_2 = 433.3582$ for cancer tissue.

Standard calculations show that $\Delta m(\alpha)$ changes its sign either for the CpGs with

$$M_{BS} > M_{oxBS} \quad \text{and} \quad \frac{M_{BS} + U_{BS}}{M_{oxBS} + U_{oxBS}} > \frac{M_{BS}}{M_{oxBS}} \tag{7}$$

or those with

$$M_{BS} < M_{oxBS} \quad \text{and} \quad \frac{M_{BS} + U_{BS}}{M_{oxBS} + U_{oxBS}} < \frac{M_{BS}}{M_{oxBS}}. \tag{8}$$

The inequalities above can be rewritten as

$$\frac{U_{BS}}{U_{oxBS}} > \frac{M_{BS}}{M_{oxBS}} > 1 \tag{9}$$

and

$$\frac{U_{BS}}{U_{oxBS}} < \frac{M_{BS}}{M_{oxBS}} < 1, \tag{10}$$

respectively. Note that these conditions imply the sign changes for the measure $\Delta\beta(\alpha)$ as well.

We can also compute the value $\alpha^* > 0$ for which $\Delta m(\alpha^*) = 0$ and $\Delta m(\alpha)$ changes its sign at α^*. Indeed, from (3) it follows that $\Delta m(\alpha^*) = 0$ for

$$\alpha^* = \frac{M_{oxBS}U_{BS} - M_{BS}U_{oxBS}}{M_{BS} - M_{oxBS}}, \tag{11}$$

which is exactly the same α^* at which $\Delta\beta(\alpha)$ changes its sign.

Relation between the subsets $\{\Delta m(\alpha) > 0\}$ and $\{\Delta m^\infty > 0\}$

Let us first address the possible relation between the values of the 5hmC measures Δm^∞ and $\Delta m(\alpha)$. In the context of a given CpG and sample, simple calculations, based on the convergence result for $\Delta m(\alpha)$, show that, for

$$U_{oxBS} \geq U_{BS},$$

the value of $\Delta m(\alpha)$ will be at least as large as the value of Δm^∞, for any $\alpha > 0$. Otherwise we will get $\Delta m(\alpha) < \Delta m^\infty$; see Fig B for an illustration of this result.

Further, let us show analytically that, for a given sample and increasing $\alpha > 0$, the subset of CpGs satisfying $\Delta m^\infty > 0$ will (approximately) become a "limiting" subset for a sequence of subsets of CpGs satisfying $\Delta m(\alpha) > 0$.

First, let us assume that $\Delta m^\infty > 0$ holds for a given sample and CpG and show that for $\alpha > 0$ large enough, the condition $\Delta m(\alpha) > 0$ will hold for this sample and CpG as well. The condition $\Delta m^\infty > 0$ evidently leads to $M_{BS} > M_{oxBS}$. On the other hand, while considering the second term in the expression

$$\Delta m(\alpha) = \log_2 \frac{M_{BS}}{M_{oxBS}} + \log_2 \frac{U_{oxBS} + \alpha}{U_{BS} + \alpha},$$

we state that

$$\forall \epsilon > 0 \text{ exists an } \alpha_0 > 0 \text{ such that } \left| \log_2 \frac{U_{oxBS} + \alpha_0}{U_{BS} + \alpha_0} \right| < \epsilon. \tag{12}$$

Thus for all $\alpha > \alpha_0$ we will get $\Delta m(\alpha) > 0$.

Conversely, let us assume that $\Delta m(\alpha) > 0$ for all $\alpha > 0$ large enough. Then, with (12), the inequality

$$\log_2 \frac{M_{BS}}{M_{oxBS}} \geq 0$$

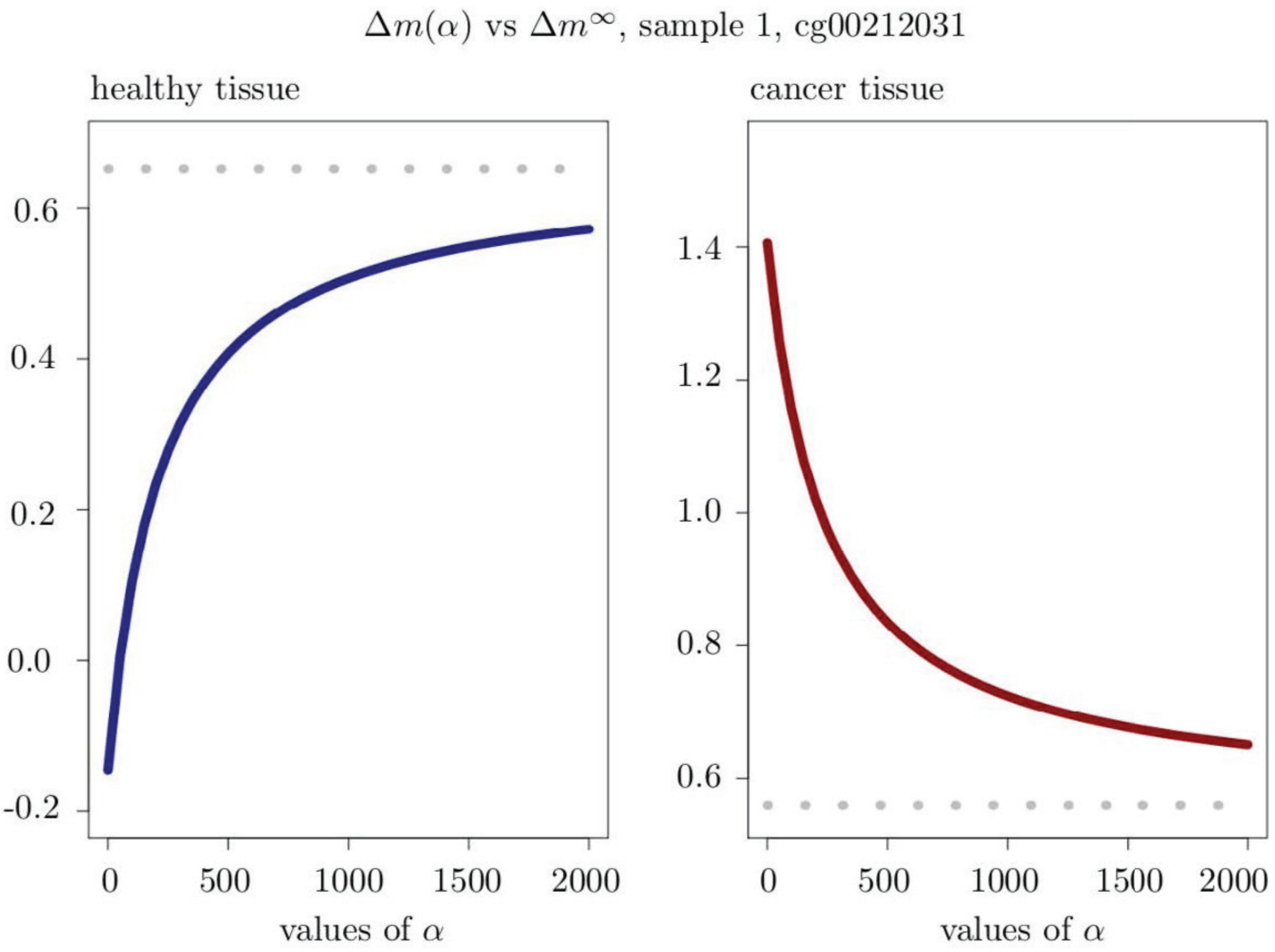

Fig B. Relation between the 5hmC measures $\Delta m(\alpha)$ and Δm^{∞}. Relation between the 5hmC measures $\Delta m(\alpha)$ (solid curves) and Δm^{∞} (dashed grey horizontal lines) for $U_{oxBS} > U_{BS}$ (the right-hand panel) as well as for $U_{oxBS} < U_{BS}$ (the left-hand panel).

must hold. This latter expression implies either $M_{BS} > M_{oxBS}$, and, as a result, $\Delta m^{\infty} > 0$, or $M_{BS} = M_{oxBS}$. As a reminder: for $M_{BS} = M_{oxBS}$ the condition $\Delta m(\alpha) > 0$ will hold only if $U_{BS} < U_{oxBS}$. But, due to our previous discussion, we agreed to ignore the set $\{M_{BS} = M_{oxBS}, U_{BS} < U_{oxBS}\}$ as irrelevant for the 5hmC quantification.

Similar considerations show that in cases when $\alpha > 0$ increases, the subset of CpGs satisfying $\Delta m(\alpha) > 0$ over all given samples (approximately) approaches the subset of CpGs which satisfy $\Delta m^{\infty} > 0$ over all given samples.

Relation between the subsets $\{\Delta h > 0\}$, $\{\Delta\beta(\alpha) > 0\}$ and $\{\Delta m^{\infty} > 0\}$

Let us analyse whether the positivity of the 5hmC measure Δh implies the positivity of two other 5hmC measures, $\Delta\beta(\alpha)$ and Δm^{∞}. First, the inequality $\Delta h > 0$ holds for all CpGs with

$$M_{BS} + U_{BS} > M_{oxBS} + U_{oxBS}.$$

Further, for $M_{BS} + U_{BS} > M_{oxBS} + U_{oxBS}$ and $M_{BS} > M_{oxBS}$ satisfied simultaneously, both 5hmC measures Δh and Δm^{∞} will take positive values.

For CpGs satisfying

$$M_{BS} + U_{BS} > M_{oxBS} + U_{oxBS},\ M_{BS} > M_{oxBS},\ \text{and}\ U_{BS} \le U_{oxBS} \tag{13}$$

simultaneously, i.e., those with $\Delta h > 0$ and $\Delta m^{\infty} > 0$, the 5hmC measure $\Delta\beta(\alpha)$ will be positive for any $\alpha > 0$. However, if in (13) the condition $U_{BS} > U_{oxBS}$ holds instead of $U_{BS} \le U_{oxBS}$, then the sign of $\Delta\beta(\alpha)$ will depend on the choice of α. In particular, we will get $\Delta\beta(\alpha) > 0$ for $\alpha > \alpha^*$, with α^* as in (11), and $\Delta\beta(\alpha) < 0$ otherwise.

For CpGs satisfying

$$M_{BS} + U_{BS} > M_{oxBS} + U_{oxBS} \quad \text{and} \quad M_{BS} \le M_{oxBS} \quad \text{and} \quad U_{BS} > U_{oxBS}$$

simultaneously, we will obviously get $\Delta h > 0$, but the 5hmC measures Δm^{∞} and $\Delta\beta(\alpha)$ will take non-positive values.
