# Supplementary material for: Statistical methods for classification of 5hmC levels based on the Illumina Inifinium HumanMethylation450 (450k) array data, under the paired bisulfite (BS) and oxidative bisulfite (oxBS) treatment
Source: PLoS One. 2019 Jun 13;14(6):e0218103. doi: 10.1371/journal.pone.0218103 (PMC6563990; doi:10.1371/journal.pone.0218103)
Supplement: S3 Appendix — Prevalence of positive results, joint prevalence of positive results, similarity analyses, relative accuracy analyses (relative sensitivity and specificity). (PDF) [file pone.0218103.s003.pdf]

Statistical methods for classification of 5hmC levels based on the Illumina Inifinium HumanMethylation450 (450k) array data, under the paired bisulfite (BS) and oxidative bisulfite (oxBS) treatment.

S3 Appendix: On the resemblance of $\Delta\beta(\alpha)$, Δm^{∞} and Δh: numerical results

Alla Slynko[1], Axel Benner[2]

June 1, 2019

Prevalence of positive results

When searching for a reduction of 5hmC levels in cancer tissue compared to the normal one in the context of the prevalence of positive results, a sample-wise analysis confirmed this anticipation only for the 5hmC measure $\Delta\beta(100)$ (paired Wilcoxon test; $p < 0.001$, the sample estimate for the pseudomedian 0.05). Further, a CpG-wise analysis also showed a significant reduction in 5hmC levels as computed on cancer tissue for the 5hmC measures $\Delta\beta(100)$ (paired Wilcoxon test; $p < 0.001$, the sample estimate for the pseudomedian 0.05) and Δm^{∞} (paired Wilcoxon test; $p < 0.001$, the sample estimate for the pseudomedian 0.01). For the measure Δh, prevalence of positive results was significantly lower on healthy tissue compared to cancer one (paired Wilcoxon test; $p < 0.001$, the sample estimate for the pseudomedian -0.04).

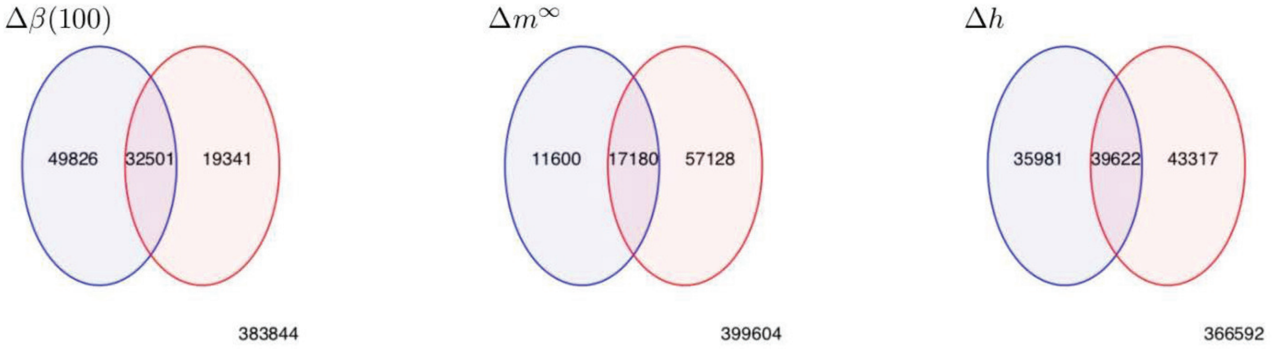

Fig A. The prevalence of positive results for each considered 5hmC measure: dendograms. The number of substantially hydroxymethylated CpGs as identified by each 5hmC measure, on both tissues and across all 38 samples. The blue color denotes healthy tissue, the red one cancer tissue. A CpG site is considered to be substantially hydroxymethylated under a given 5hmC measure x, if at least 75 % of all values of x computed on this CpG and across all 38 samples are positive.

[1]Department of Statistics and Actuarial Science, University of Waterloo, Waterloo, Canada, alla.a.slynko@gmail.com

[2]Division of Biostatistics, German Cancer Research Center, Heidelberg, Germany

Conservativeness

When comparing prevalences of positive results of any two 5hmC measure on a given tissue, a sample-wise analysis resulted in the 5hmC measure Δm^{∞} being less conservative than Δh on healthy tissue (the paired Wilcoxon test; $p = 0.003$, the sample estimate for the pseudomedian 0.05). On cancer tissue, the 5hmC measure Δm^{∞} appeared to be less conservative than $\Delta\beta(100)$ (the paired Wilcoxon test; $p = 0.049$, the sample estimate for the pseudomedian 0.07).

The same analysis, performed CpG-wise, determined Δm^{∞} as being the least conservative 5hmC measure, on both considered tissues. Further, on healthy tissue $\Delta\beta(100)$ appeared to be less conservative compared to Δh (the paired Wilcoxon test; $p < 0.001$, the sample estimate for the pseudomedian 0.03); on cancer tissue, Δh was less conservative than $\Delta\beta(100)$ (the paired Wilcoxon test; $p < 0.001$, the sample estimate for the pseudomedian 0.05).

Joint prevalence of positive results

A sample-wise analysis revealed the joint prevalence of the measures $\Delta\beta(100)$ and Δm^{∞} exceeding the joint prevalence of $\Delta\beta(100)$ and Δh significantly, both on healthy (the paired Wilcoxon test; $p < 0.001$, the sample estimate for the pseudomedian 0.13) and cancer (the paired Wilcoxon test; $p < 0.001$, the sample estimate for the pseudomedian 0.11) tissue. The same result holds for the joint prevalences of the measures Δm^{∞} and Δh as well as of the measures $\Delta\beta(100)$ and Δh, both on healthy (the paired Wilcoxon test; $p < 0.001$, the sample estimate for the pseudomedian -0.15) and cancer (the paired Wilcoxon test; $p < 0.001$, the sample estimate for the pseudomedian -0.18) tissue. Finally, on cancer tissue, the joint prevalence of the measures $\Delta\beta(100)$ and Δm^{∞} significantly exceeded the joint prevalence of Δm^{∞} and Δh (the paired Wilcoxon test; $p = 0.006$, the sample estimate for the pseudomedian -0.07).

A CpG-wise analysis of joint prevalences revealed the joint prevalence of the measures $\Delta\beta(100)$ and Δm^{∞} on healthy tissue being significantly higher than the corresponding joint prevalence on cancer tissue (the paired Wilcoxon test; $p < 0.001$, the sample estimate for the pseudomedian 0.04); similar result is true for the joint prevalence of the measures $\Delta\beta(100)$ and Δh (the paired Wilcoxon test; $p < 0.001$, the sample estimate for the pseudomedian 0.03). On the other hand, the joint prevalence of $\Delta\beta(100)$ and Δm^{∞} is significantly lower than the joint prevalence of the measures Δm^{∞} and Δh, both on healthy (the paired Wilcoxon test; $p < 0.001$, the sample estimate for the pseudomedian -0.03) and on cancer tissue (the paired Wilcoxon test; $p < 0.001$, the sample estimate for the pseudomedian -0.08).

Similarity analyses

While performing the similarity analysis for each given sample, the 5hmC measure $\Delta\beta(100)$ was less similar to Δh than to Δm^{∞}, both on healthy (the paired Wilcoxon test; $p < 0.001$, the sample estimate for the pseudomedian -0.21) and cancer (the paired Wilcoxon test; $p < 0.001$, the sample estimate for the pseudomedian -0.21) tissue.

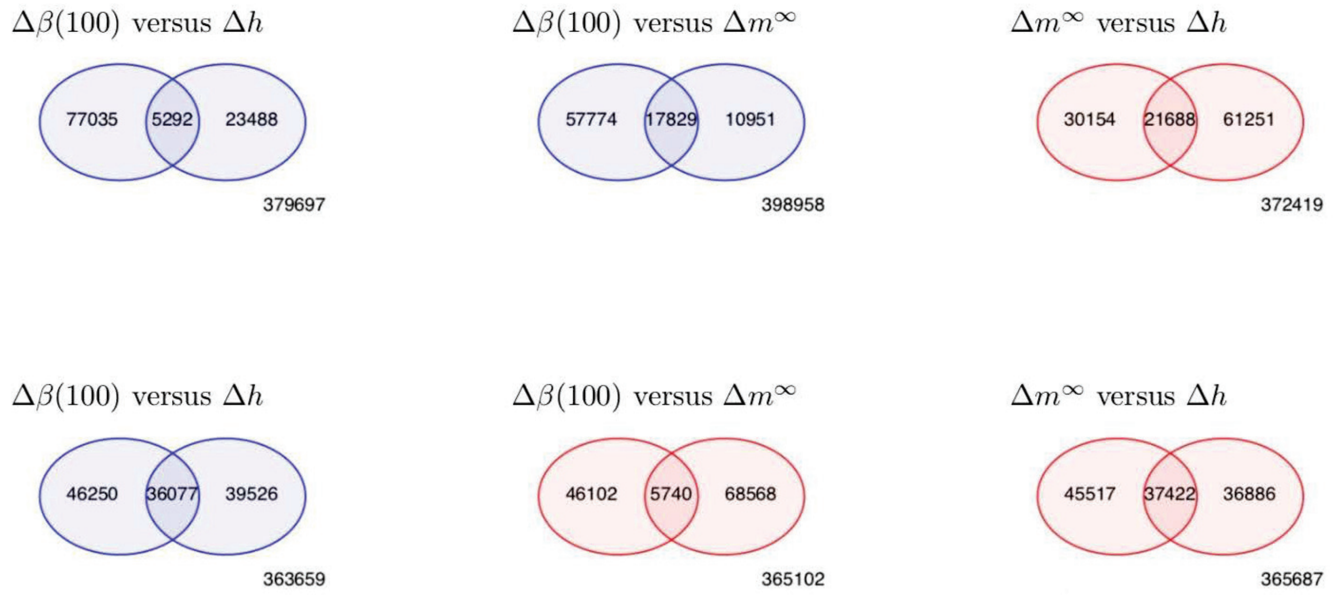

Fig B. The joint prevalence of positive results for any two considered 5hmC measures: dendograms. The number of substantially hydroxymethylated CpGs as identified simultaneously by any two 5hmC measures, on healthy (three upper panels) and cancer (three lower panels) tissues and across all 38 samples. A CpG site is considered to be substantially hydroxymethylated under two given 5hmC measures x_1 and x_2, if on this CpG both measures take positive values simultaneously on at least 75 % of all 38 samples.

Relative accuracy analyses

Relative sensitivity

While checking for a significant difference in relative sensitivities as computed on healthy and cancer tissue, a sample-wise analysis revealed such difference for the relative sensitivities $SE_r(\Delta\beta(100)\,|\,\Delta h)$ (the paired Wilcoxon test; $p = 0.005$, the sample estimate for the pseudomedian 0.04) and $SE_r(\Delta\beta(100)\,|\,\Delta m^{\infty})$ (the paired Wilcoxon test; $p = 0.021$, the sample estimate for the pseudomedian 0.06). In the same sample-wise analysis, performed on healthy tissue, the 5hmC measure Δm^{∞} demonstrated a stronger sensitivity with respect to Δh than Δh did with respect to Δm^{∞} (the paired Wilcoxon test; $p = 0.001$, the sample estimate for the pseudomedian 0.11). Further, there was a trend for a significant increase in the relative sensitivity $SE_r(\Delta m^{\infty}\,|\,\Delta\beta(100))$ compared to the relative sensitivity $SE_r(\Delta\beta(100)\,|\,\Delta m^{\infty})$ on cancer tissue (the paired Wilcoxon test; $p = 0.083$, the sample estimate for the pseudomedian 0.08).

A CpG-wise analysis of relative sensitivities revealed the 5hmC measure $\Delta\beta(100)$ being less sensitive with respect to the measure Δh on cancer tissue than the other way around (the paired Wilcoxon test; $p < 0.001$, the sample estimate for the pseudomedian -0.04). This situation changed to the opposite on healthy tissue (the paired Wilcoxon test; $p < 0.001$, the sample estimate for the pseudomedian 0.03). Further, the measure Δm^{∞} showed a stronger sensitivity with respect to the measure $\Delta\beta(100)$ than $\Delta\beta(100)$ did with respect to Δm^{∞}, both on healthy (the paired Wilcoxon test; $p < 0.001$, the

sample estimate for the pseudomedian 0.03) and cancer (the paired Wilcoxon test; $p < 0.001$, the sample estimate for the pseudomedian 0.08) tissue. Analogous result holds for the measures Δm^{∞} and Δh, with $SE_r(\Delta m^{\infty} \,|\, \Delta h)$ exceeding $SE_r(\Delta h \,|\, \Delta m^{\infty})$ both on healthy (the paired Wilcoxon test; $p < 0.001$, the sample estimate for the pseudomedian 0.08) and cancer (the paired Wilcoxon test; $p < 0.001$, the sample estimate for the pseudomedian 0.02) tissue.

Relative specificity

While analyzed sample-wise for its relative specificity, the measure $\Delta\beta(100)$ demonstrated a significantly weaker specificity with respect to Δh on healthy tissue than on cancer tissue (the paired Wilcoxon test; $p = 0.01$, the sample estimate for the pseudomedian -0.03); similar result holds for relative specificity of the measure Δm^{∞} with respect to the measure Δh (the paired Wilcoxon test; $p = 0.034$, the sample estimate for the pseudomedian -0.05). The same analysis, while performed CpG-wise, showed all relative specificities differentiating significantly between healthy and cancer tissue. Further, the 5hmC measure $\Delta\beta(100)$ demonstrated a stronger specificity with respect to the measure Δm^{∞} than Δm^{∞} did with respect to $\Delta\beta(100)$, both on healthy (the paired Wilcoxon test; $p = 0.054$, the sample estimate for the pseudomedian 0.14) as well as on cancer (the paired Wilcoxon test; $p = 0.008$, the sample estimate for the pseudomedian 0.16) tissue, with the difference being more substantial on cancer tissue.

In a CpG-wise analysis, performed on healthy tissue, the measure $\Delta\beta(100)$ demonstrated a significantly weaker specificity with respect to the measure Δh than Δh did with respect to $\Delta\beta(100)$ (the paired Wilcoxon test; $p < 0.001$, the sample estimate for the pseudomedian -0.03); this situation changes to the opposite while considering the same relative specificities on cancer tissue (the paired Wilcoxon test; $p < 0.001$, the sample estimate for the pseudomedian 0.04). Further, the measure Δm^{∞} showed a weaker specificity with respect to $\Delta\beta(100)$ than $\Delta\beta(100)$ did with respect to Δm^{∞}, both on healthy (the paired Wilcoxon test; $p < 0.001$, the sample estimate for the pseudomedian -0.04) as well as on cancer tissue (the paired Wilcoxon test; $p < 0.001$, the sample estimate for the pseudomedian -0.09). Similar result is true for the measures Δm^{∞} and Δh, both on healthy (the paired Wilcoxon test; $p < 0.001$, the sample estimate for the pseudomedian -0.12) and on cancer tissue (the paired Wilcoxon test; $p < 0.001$, the sample estimate for the pseudomedian -0.04).
