# Supplementary material for: Statistical methods for classification of 5hmC levels based on the Illumina Inifinium HumanMethylation450 (450k) array data, under the paired bisulfite (BS) and oxidative bisulfite (oxBS) treatment
Source: PLoS One. 2019 Jun 13;14(6):e0218103. doi: 10.1371/journal.pone.0218103 (PMC6563990; doi:10.1371/journal.pone.0218103)
Supplement: S4 Appendix — (PDF) [file pone.0218103.s004.pdf]

Statistical methods for classification of 5hmC levels based on the Illumina Inifinium HumanMethylation450 (450k) array data, under the paired bisulfite (BS) and oxidative bisulfite (oxBS) treatment.

S4 Appendix: A comparison of the 5hmC measures $\Delta\beta(\alpha), \Delta h$ and Δm^{∞} with the oxBS-MLE and OxyBS procedures

Alla Slynko[1], Axel Benner[2] June 1, 2019

In this section we compare the results on the 5hmC detection as provided by the 5hmC measures $\Delta\beta(100), \Delta h$ and Δm^{∞} with the analogous results derived from the oxBS-MLE and OxyBS procedures introduced in [44, 47]; all analyses were performed sample-wise, on both healthy and cancer tissue. The estimates provided by the oxBS-MLE and OxyBS procedures are almost identical in case of a screening procedure. Thus, we compare our 5hmC measures with the oxBS-MLE only, since this procedure has a much shorter computation time compared to that of the OxyBS procedure [47].

We start our analysis with the following proposition:

Proposition: *In a screening procedure, the 5hmC measure $\Delta\beta(0)$ will flag the same CpGs as being hydroxymethylated as the oxBS-MLE (and OxyBS procedure) will do.*

Proof: We only show that all CpGs with $\Delta\beta(0) > 0$ will be flagged as hydroxymethylated by both oxBS-MLE and OxyBS procedures. The converse implication can be obtained in a similar way.

For a given CpG and sample, let $\Delta\beta(0) > 0$. Then we get

$$\frac{M_{BS}}{M_{BS} + U_{BS}} > \frac{M_{oxBS}}{M_{oxBS} + U_{oxBS}}.$$

With this inequality, due to Supplementary material provided in [47], the oxBS-MLE procedure provides an estimate for the 5hmC proportion $\hat{\pi}_{5hmC} > 0$. Moreover, the estimate $\hat{\pi}_{5hmC}$ is equal to $\Delta\beta(0)$.

Further, with the notations of [44] and, in particular, with the unmethylated proportion π_1 and 5mC

[1]Department of Statistics and Actuarial Science, University of Waterloo, Waterloo, Canada, alla.a.slynko@gmail.com

[2]Division of Biostatistics, German Cancer Research Center, Heidelberg, Germany

proportion π_2, $\Delta\beta(0) > 0$ implies

$$\frac{M_{BS}}{M_{BS}+U_{BS}} > \frac{M_{oxBS}}{M_{oxBS}+U_{oxBS}} \Longleftrightarrow$$

$$\frac{M_{BS}}{M_{BS}+U_{BS}} > \pi_2 \Longleftrightarrow \frac{(M_{BS}+U_{BS})-U_{BS}}{M_{BS}+U_{BS}} > \pi_2 \Longleftrightarrow$$

$$1-\pi_1 > \pi_2 \Longleftrightarrow \pi_1+\pi_2 < 1$$

and thus, due to $\pi_1+\pi_2+\pi_3 = 1$, the 5hmC proportion π_3 must be positive. This proves our result.

Now we describe our observations when comparing the proposed 5hmC measures with the oxBS-MLE procedure on healthy and cancer tissue numerically.

Prevalence of positive results: To determine the prevalence of positive results in terms of the oxBS-MLE procedure, we consider all CpGs with the 5hmC level larger than 0.001% as being hydroxymethylated; this threshold was suggested in the documentation to the package *OxyBS* as "small positive value representing numerical zero" [44].

First, we observe the prevalence of positive results provided by the oxBS-MLE to be higher in healthy tissue compared to cancer tissue (the paired Wilcoxon test; $p < 0.001$, the sample estimate for the pseudomedian 0.045). This result corresponds to the statement on a reduction of 5hmC levels in cancer tissue presented in [24]. Further, the measure $\Delta\beta(100)$ provides a higher prevalence of positive results than the oxBS-MLE procedure, both on healthy (the paired Wilcoxon test; $p < 0.001$, the sample estimate for the pseudomedian < 0.001) and cancer tissue (the paired Wilcoxon test; $p < 0.001$, the sample estimate for the pseudomedian < 0.001). On cancer tissue, the measure Δm^{∞} demonstrates a higher prevalence of positive results than the oxBS-MLE procedure does (the paired Wilcoxon test; $p = 0.049$, the sample estimate for the pseudomedian 0.072). For all other comparisons, no significant differences were observed.

Joint prevalence of positive results: When analyzing the joint prevalence of positive results as estimated for a given 5hmC measure and the oxBS-MLE procedure, we observe that this joint prevalence is the highest for the measure $\Delta\beta(100)$, followed by the measure Δm^{∞}, both on healthy and cancer tissue. In particular, the joint prevalence of positive results of $\Delta\beta(100)$ and the oxBS-MLE exceeds the corresponding joint prevalence of Δm^{∞} and the oxBS-MLE on healthy (the paired Wilcoxon test; $p < 0.001$, the sample estimate for the pseudomedian 0.118) as well as on cancer tissue (the paired Wilcoxon test; $p < 0.001$, the sample estimate for the pseudomedian 0.107). Further, the joint prevalence of positive results of Δm^{∞} and the oxBS-MLE exceeds the corresponding joint prevalence of Δh and the oxBS-MLE on healthy (the paired Wilcoxon test; $p < 0.001$, the sample estimate for the pseudomedian 0.129) as well as on cancer tissue (the paired Wilcoxon test; $p < 0.001$, the sample estimate for the pseudomedian 0.107).

Similarity analyses: In terms of the similarity measure $\mathbb{S}$, the 5hmC measure $\Delta\beta(100)$ exhibits a stronger similarity to the oxBS-MLE procedure on healthy tissue than it does on cancer tissue

(the paired Wilcoxon test; $p = 0.0015$, the sample estimate for the pseudomedian < 0.001). In total, the measure $\Delta\beta(100)$ shows the strongest similarity to the oxBS-MLE procedure, both on healthy (the paired Wilcoxon test; $p < 0.001$, the sample estimate for the pseudomedian 0.297) and on cancer tissue (the paired Wilcoxon test; $p < 0.001$, the sample estimate for the pseudomedian 0.299). Further, the measure Δm^{∞} demonstrates a stronger similarity to the oxBS-MLE procedure than the measure Δh, both on healthy (the paired Wilcoxon test; $p < 0.001$, the sample estimate for the pseudomedian 0.21) as well as on cancer tissue (the paired Wilcoxon test; $p < 0.001$, the sample estimate for the pseudomedian 0.212).
