# Supplementary material for: Statistical methods for classification of 5hmC levels based on the Illumina Inifinium HumanMethylation450 (450k) array data, under the paired bisulfite (BS) and oxidative bisulfite (oxBS) treatment
Source: PLoS One. 2019 Jun 13;14(6):e0218103. doi: 10.1371/journal.pone.0218103 (PMC6563990; doi:10.1371/journal.pone.0218103)
Supplement: S5 Appendix — (PDF) [file pone.0218103.s005.pdf]

Statistical methods for classification of 5hmC levels based on the Illumina Inifinium HumanMethylation450 (450k) array data, under the paired bisulfite (BS) and oxidative bisulfite (oxBS) treatment.

S5 Appendix: A comparison of numerical analyses performed on raw and normalized data

Alla Slynko[1], Axel Benner[2] June 1, 2019

In this section we check some particular numerical results obtained sample-wise on raw data against the analogous results based on normalized data. Data were normalized using three different normalization procedures, the *funNorm*, *SWAN* and *Illumina* procedures, available in the *R*-package *minfi* [51].

Prevalence of positive results

Here we compare the prevalences of positive results estimated sample-wise on raw and normalized data. First, Table 1 (mostly) confirms a reduction of the 5hmC levels in cancer tissue compared to healthy one. In particular, in terms of the 5hmC measure $\Delta\beta(100)$, such reduction was observed both on raw and normalized data. On the other hand, in terms of the measure Δm^{∞}, only two normalized data sets, *funNorm* and *Illumina* data sets, confirmed the above statement about a reduction of the 5hmC levels. Finally, for the measure Δh, no significant reduction of the 5hmC levels in cancer tissue was detected, neither on raw nor on normalized data.

Further, when comparing the sample-wise prevalence of positive results of $\Delta\beta(100)$ on raw and normalized data, no significant difference in such prevalences on healthy tissue was observed. On cancer tissue, prevalence of positive results of $\Delta\beta(100)$ estimated on raw data exceeded the corresponding prevalence estimated on *SWAN* data (paired Wilcoxon test; $p = 0.023$, the sample estimate for the pseudomedian 0.01) and on *Illumina* data (paired Wilcoxon test; $p = 0.041$, the sample estimate for the pseudomedian 0.02). In terms of the 5hmC measure Δm^{∞}, the prevalence of positive results estimated on raw data always exceeded the corresponding prevalence estimated on normalized data, both on healthy and cancer tissue. This result is also true in terms of the measure Δh, although only on cancer tissue.

On healthy tissue, the prevalence of positive results estimated on *funNorm* data is significantly lower compared to the corresponding prevalence estimated on *Illumina* data, both for the measure Δm^{∞} (paired Wilcoxon test; $p = 0.006$, the sample estimate for the pseudomedian -0.04) and for Δh (paired Wilcoxon test; $p = 0.021$, the sample estimate for the pseudomedian -0.04).

[1]Department of Statistics and Actuarial Science, University of Waterloo, Waterloo, Canada, alla.a.slynko@gmail.com

[2]Division of Biostatistics, German Cancer Research Center, Heidelberg, Germany

	p-value	pm
raw data	< 0.001	0.04
funNorm data	0.005	0.03
SWAN data	< 0.001	0.05
Illumina data	< 0.001	0.05

(a)

	p-value	pm
raw data	0.640	-0.01
funNorm data	0.011	0.07
SWAN data	0.387	-0.01
Illumina data	< 0.001	0.07

(b)

	p-value	pm
raw data	0.875	-0.06
funNorm data	0.171	0.03
SWAN data	0.150	-0.06
Illumina data	0.068	0.04

(c)

Table 1. On a reduction of the prevalence of positive results in cancer tissue. Analyses were performed sample-wise on raw and normalized data, by means of the paired Wilcoxon signed rank test; all three 5hmC measure, $\Delta\beta$ (the panel (a)), Δm^{∞} (the panel (b)) and Δh (the panel (c)) were considered. The p-values in the tables correspond to the p-values provided by the applied test, the pm-values are the corresponding pseudomedian estimates. For instance, the first row of the table (a) shows a significant difference in the prevalence of positive results of $\Delta\beta$ on healthy and cancer tissue (the p-value< 0.001), when estimated on raw data; the corresponding pseudomedian estimate is equal to 0.04. On the other hand, the first row of the table (b) shows no significant difference in the prevalence of positive results of Δm^{∞} on healthy and cancer tissue, when estimated on raw data (the p-value > 0.05).

On cancer tissue, the prevalence of positive results of $\Delta\beta(100)$ estimated on *funNorm* data exceeds the corresponding prevalence estimated on *Illumina* data (paired Wilcoxon test; $p = 0.002$, the sample estimate for the pseudomedian 0.03). In terms of Δm^{∞}, the prevalence of positive results estimated on *SWAN* data is the highest one, compared to the corresponding prevalences estimated on *funNorm* data (paired Wilcoxon test; $p = 0.001$, the sample estimate for the pseudomedian 0.13) as well as on *Illumina* data (paired Wilcoxon test; $p = 0.021$, the sample estimate for the pseudomedian 0.1). This result remains true for the 5hmC measure Δh as well.

Similarity analyses

Due to our previous discussion, pairwise similarity between any two 5hmC measures can be characterized by means of the similarity coefficient $\mathbb{S}$. To check for a possible tissue dependence in similarities among the considered 5hmC measures, we compared the pairwise similarities on healthy and cancer tissue. The results are presented in Table 2. As that table shows, both on *funNorm* and *Illumina* data, similarities described in terms of $\mathbb{S}(\Delta\beta(100), \Delta m^{\infty})$ on healthy tissue exceed the corresponding similarities on cancer tissue; analogous result holds for similarities measured by $\mathbb{S}(\Delta\beta(100), \Delta h)$. On *Illumina* data set, similarity measured by $\mathbb{S}(\Delta h, \Delta m^{\infty})$ on cancer tissue exceeds the analogous similarity on healthy tissue (paired Wilcoxon test; $p = 0.009$, the sample estimate for

	p-value	pm
raw data	0.745	0.01
funNorm data	0.001	0.03
SWAN data	0.206	0.01
Illumina data	< 0.001	0.03

(a)

	p-value	pm
raw data	0.318	0.01
funNorm data	< 0.001	0.03
SWAN data	0.232	0.01
Illumina data	0.001	0.02

(b)

	p-value	pm
raw data	0.546	-0.002
funNorm data	0.382	0.002
SWAN data	0.432	0.003
Illumina data	0.009	-0.011

(c)

Table 2. On pairwise similarity among three considered 5hmC measures, as estimated on healthy and cancer tissue. Analyses were performed sample-wise on raw and normalized data, by means of the paired Wilcoxon signed rank test; in terms of the similarity coefficient $\mathbb{S}$, the quantities $\mathbb{S}(\Delta\beta(100), \Delta m^{\infty})$ (the panel (a)), $\mathbb{S}(\Delta\beta(00), \Delta h)$ (the panel (b)) and $\mathbb{S}(\Delta h, \Delta m^{\infty})$ (the panel (c)) were analyzed. The p-values in the tables correspond to the p-values provided by the applied test, the pm-values are the corresponding pseudomedian estimates.

the pseudomedian -0.01). Altogether, when comparing pairwise similarities for three considered 5hmC measures on healthy tissue, we observe the following relation

$$\mathbb{S}(\Delta m^{\infty}, \Delta h) > \mathbb{S}(\Delta\beta(100), \Delta m^{\infty}) > \mathbb{S}(\Delta\beta(100), \Delta h),$$

both on raw and normalized data. Analogous result holds on cancer tissue.

When comparing similarity coefficients as estimated on three different normalized data sets, *funNorm*, *SWAN* and *Illumina*, no difference in similarities described by the coefficient $\mathbb{S}(\Delta\beta(100), \Delta h)$, both on healthy and cancer tissue could be observed so far. Further, on healthy tissue, similarity characterized by $\mathbb{S}(\Delta\beta(100), \Delta m^{\infty})$ is the strongest on *funNorm* data set (paired Wilcoxon test; $p = 0.001$, the sample estimate for the pseudomedian 0.03), followed by the similarity estimated on *SWAN* data set (paired Wilcoxon test; $p < 0.001$, the sample estimate for the pseudomedian -0.036); an analogous result (with different p-values and pseudomedians) holds on cancer tissue as well. As for similarity characterized by $\mathbb{S}(\Delta h, \Delta m^{\infty})$, we observed that on healthy tissue this similarity is the strongest on *SWAN* data; on cancer tissue, this similarity is the strongest on *Illumina* data set.
